# Identification of a Novel Regulator of Clostridioides difficile Cortex Formation

**DOI:** 10.1128/mSphere.00211-21

**Published:** 2021-05-28

**Authors:** Megan H. Touchette, Hector Benito de la Puebla, Carolina Alves Feliciano, Benjamin Tanenbaum, Monica Schenone, Steven A. Carr, Aimee Shen

**Affiliations:** a Department of Molecular Biology and Microbiology, Tufts University School of Medicine, Boston, Massachusetts, USA; b Broad Institute of MIT and Harvard, Cambridge, Massachusetts, USA; University of Iowa

**Keywords:** *Clostridioides difficile*, coat, cortex, spore assembly, sporulation

## Abstract

Clostridioides difficile is a leading cause of health care-associated infections worldwide. These infections are transmitted by C. difficile′s metabolically dormant, aerotolerant spore form. Functional spore formation depends on the assembly of two protective layers, a thick layer of modified peptidoglycan known as the cortex layer and a multilayered proteinaceous meshwork known as the coat. We previously identified two spore morphogenetic proteins, SpoIVA and SipL, that are essential for recruiting coat proteins to the developing forespore and making functional spores. While SpoIVA and SipL directly interact, the identities of the proteins they recruit to the forespore remained unknown. Here, we used mass spectrometry-based affinity proteomics to identify proteins that interact with the SpoIVA-SipL complex. These analyses identified the *Peptostreptococcaceae* family-specific, sporulation-induced bitopic membrane protein CD3457 (renamed SpoVQ) as a protein that interacts with SipL and SpoIVA. Loss of SpoVQ decreased heat-resistant spore formation by ∼5-fold and reduced cortex thickness ∼2-fold; the thinner cortex layer of Δ*spoVQ* spores correlated with higher levels of spontaneous germination (i.e., in the absence of germinant). Notably, loss of SpoVQ in either *spoIVA* or *sipL* mutants prevented cortex synthesis altogether and greatly impaired the localization of a SipL-mCherry fusion protein around the forespore. Thus, SpoVQ is a novel regulator of C. difficile cortex synthesis that appears to link cortex and coat formation. The identification of SpoVQ as a spore morphogenetic protein further highlights how *Peptostreptococcaceae* family-specific mechanisms control spore formation in C. difficile.

**IMPORTANCE** The Centers for Disease Control has designated Clostridioides difficile as an urgent threat because of its intrinsic antibiotic resistance. C. difficile persists in the presence of antibiotics in part because it makes metabolically dormant spores. While recent work has shown that preventing the formation of infectious spores can reduce C. difficile disease recurrence, more selective antisporulation therapies are needed. The identification of spore morphogenetic factors specific to C. difficile would facilitate the development of such therapies. In this study, we identified SpoVQ (CD3457) as a spore morphogenetic protein specific to the *Peptostreptococcaceae* family that regulates the formation of C. difficile’s protective spore cortex layer. SpoVQ acts in concert with the known spore coat morphogenetic factors, SpoIVA and SipL, to link formation of the protective coat and cortex layers. These data reveal a novel pathway that could be targeted to prevent the formation of infectious C. difficile spores.

## INTRODUCTION

The bacterial pathogen Clostridioides difficile is a leading cause of health care-associated infections worldwide. In the United States, it is responsible for an estimated ∼470,000 infections with an associated treatment cost of >$1 billion annually (1). C. difficile causes colitis and can lead to severe complications such as pseudomembranous colitis, toxic megacolon, and death, particularly in elderly populations (2). These complications are more frequent in cases of disease recurrence (3), which occurs in ∼20% of C. difficile infections (4).

Prior antibiotic usage predisposes individuals to C. difficile infection because antibiotics disrupt the endogenous microbiota that typically provides colonization resistance against C. difficile infection (5, 6). C. difficile can persist in the face of antibiotic treatment in part because its metabolically dormant spore form is inert to antibiotics. This metabolic dormancy allows C. difficile spores to survive in the presence of oxygen and transmit disease (7, 8). Accordingly, preventing spore formation can reduce C. difficile disease recurrence in mice (9).

The basic steps of C. difficile spore formation resemble those first defined in Bacillus subtilis (10, 11). Once sporulation is activated at the transcriptional level, the first morphological event of sporulation is the formation of a polar septum during asymmetric division. This event creates a larger mother cell and smaller forespore cell, both of which induce distinct transcriptional programs that drive specific morphological changes. The larger mother cell will engulf the smaller forespore in a process analogous to phagocytosis. Following engulfment, the forespore is surrounded by (i) an outer forespore membrane derived from the mother cell and (ii) an inner forespore membrane derived from the forespore. The mother cell then produces an extensive array of coat proteins that localize to and encase the forespore to form the concentric proteinaceous shells that make up the coat. This coat layer protects spores against oxidative, chemical, and enzymatic insults. The mother cell and the forespore also collaborate to synthesize a thick layer of modified peptidoglycan known as the cortex. This massive cell wall layer constrains the spore’s size and ultimately prevents water from entering the developing forespore even as the forespore imports the spore-specific small molecule, calcium dipicolinic acid. The low water content of the spore cytosol, also known as the core, impairs metabolic processes and is essential for maintaining metabolic dormancy (12, 13).

While the general morphological stages of spore formation are conserved between C. difficile, B. subtilis, and other mono-spore formers (14), the mechanisms used by these organisms to complete each morphological stage can differ significantly. For example, several spore morphogenetic proteins identified in B. subtilis have different functional requirements in C. difficile (10). These differences are particularly acute at the level of coat assembly. SpoVM is conserved across most spore formers (15) and is essential for coat assembly around both the B. subtilis forespore and for cortex synthesis (16, 17). However, SpoVM is mostly dispensable for functional spore formation in C. difficile, although ∼30% of sporulating C. difficile *spoVM* mutant cells exhibit cortex abnormalities (18). C. difficile SpoIVA phenocopies B. subtilis SpoIVA in that both are strictly required for functional spore formation and coat encasement (19–21). However, unlike B. subtilis SpoIVA, C. difficile SpoIVA is not strictly needed for cortex synthesis (20, 21).

B. subtilis SpoVM and SpoIVA are both essential for cortex formation because defects in their localization around the forespore trigger a quality control pathway that leads to mother cell lysis (17). While this quality control pathway is absent in the *Clostridia* (22), SpoVM and SpoIVA nevertheless impact cortex synthesis in C. difficile because mutants lacking either of these proteins generate forespores with cortex abnormalities (18, 23).

Beyond these conserved spore morphogenetic proteins, C. difficile uses clostridial- and *Peptostreptococcaceae* family-specific spore morphogenetic proteins to mediate spore assembly. SipL (SpoIVA interacting protein L) is a clostridial-specific spore morphogenetic protein that directly binds SpoIVA and is required for other coat proteins to localize to and encase the forespore (20). Both these proteins are made early in sporulation under the control of the mother cell-specific sigma factor, σ^E^, the first sporulation-specific sigma factor that gets activated in the mother cell (24). A C. difficile *sipL* mutant resembles a C. difficile *spoIVA* mutant in that both fail to polymerize coat layers around the forespore and produce cortex layers that are often irregular in shape and thickness (18, 20, 25). CotL is a *Peptostreptococcaceae* family-specific coat morphogenetic protein that also regulates cortex thickness (26). Loss of CotL strongly impairs the localization and/or retention of coat and cortex-localized proteins in mature spores, which also produce a thinner cortex layer. Taken together, these analyses suggest an intriguing but poorly understood link between coat and cortex assembly in C. difficile. In this study, we identify SpoVQ as a novel *Peptostreptococcaceae*-family protein that appears to link these two morphological processes. In particular, SpoVQ regulates cortex synthesis and genetically and physically interacts with the SpoIVA and SipL coat morphogenetic proteins.

## RESULTS

### Identification of SipL-binding proteins using coimmunoprecipitation.

**FIG 1:**
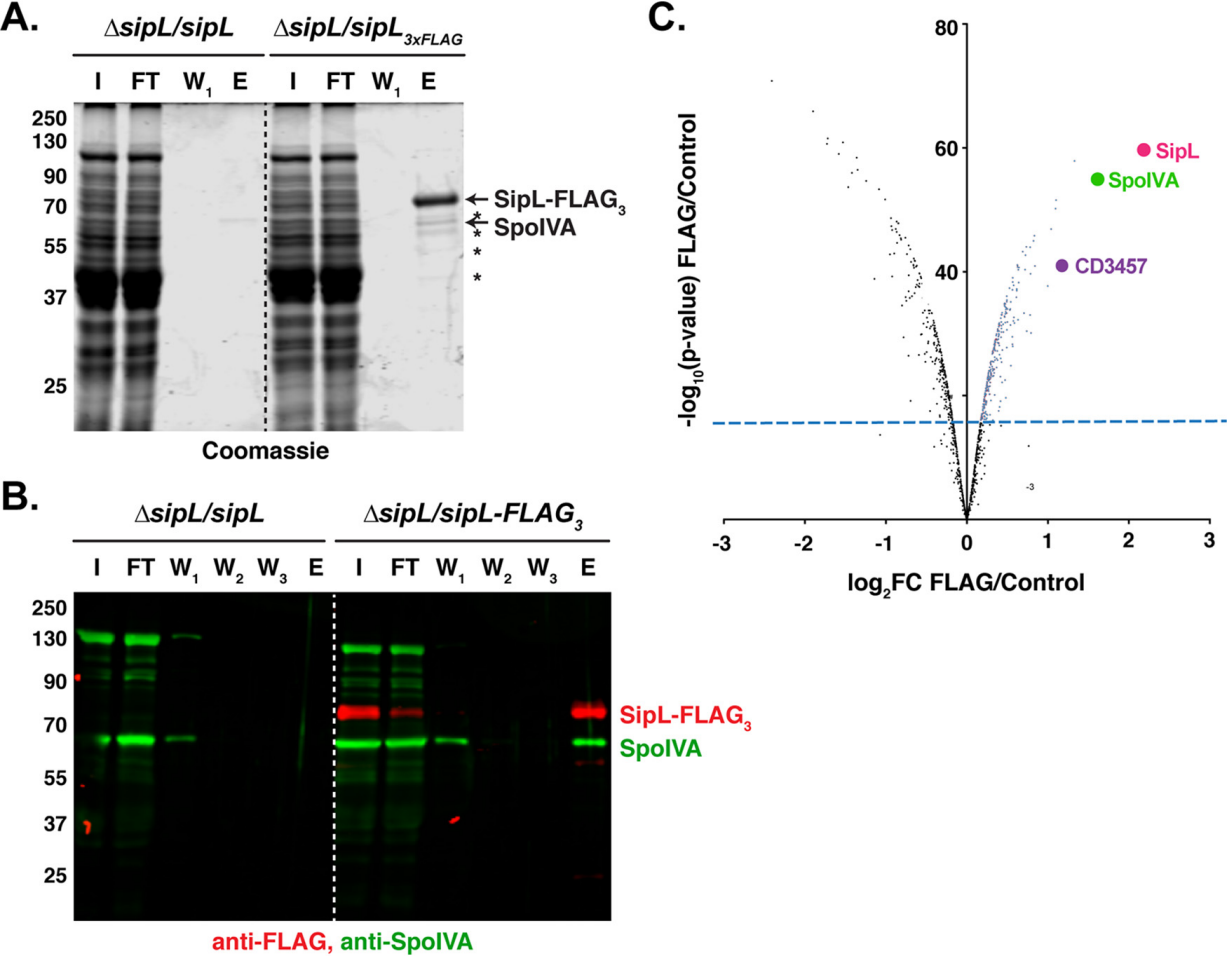
Mass spectrometry analyses of SipL coimmunoprecipitations identify SpoVQ as an interacting partner. (A) Coomassie stain of SipL coimmunoprecipitation fractions reveals potential interacting partners highlighted by asterisks. I, input; FT, flowthrough; W_1_, first wash; W_2_, second wash; W_3_, third wash; E, FLAG-peptide eluate. (B) Western blot analysis detecting SpoIVA and SipL in SipL coimmunoprecipitation fractions. SipL-FLAG_3_ was detected using an anti-FLAG antibody (red), while untagged SpoIVA was detected using an anti-SpoIVA antibody (green). (C) Volcano plot of mass spectrometry data showing enrichment of SipL-interacting proteins in red that exceeded the minimum statistical threshold of *P* < 0.01 with a fold change (FC) of ≥2.

Both SpoIVA and SipL are landmark proteins for coat morphogenesis. Loss of either of these proteins results in polymerized coat mislocalizing to the cytosol or failing to fully encase the forespore (20, 23, 25). Our previous work suggests that SpoIVA and SipL are recruited as a complex to the forespore (25) and that binding between these two proteins facilitates their encasement of the forespore (23). To identify interacting partners of SipL and potentially SpoIVA, we immunoprecipitated members of the complex and analyzed the pulldowns by quantitative mass spectrometry-based proteomics (27, 28). Specifically, we immunoprecipitated a FLAG-tagged SipL variant that we previously showed pulls down untagged SpoIVA (23). The strain producing this variant expresses a *sipL* construct encoding SipL with three tandem FLAG tags at its C terminus from the ectopic *pyrE* locus of a Δ*sipL* mutant strain (25). (The *pyrE* locus was used for all complementation strains generated in this study [29]). To determine whether proteins beyond SpoIVA could be identified in SipL-FLAG_3_ pulldowns, we Coomassie-stained FLAG peptide eluates from coimmunoprecipitations with the Δ*sipL*/*sipL-FLAG_3_* strain or untagged *sipL* complementation strain. Several bands representing potential interacting partners were observed in the elution fraction of the SipL-FLAG_3_ coimmunoprecipitation but not in the untagged SipL control sample (Fig. 1A). The band at 65 kDa is likely SpoIVA based on Western blot analyses (Fig. 1B).

To identify additional SipL-interacting proteins, we performed trypsin on-bead digestions of the coimmunoprecipitation samples and labeled the digestions with isobaric tag reagents. After equally mixing the labeled samples, the pooled sample was subjected to quantitative liquid chromatography tandem mass spectrometry (LC-MS/MS). Analyses of these samples confirmed SpoIVA as a SipL-interacting partner (3-fold enrichment, *P* < 10^−6^) and identified a number of cytosolic proteins (Fig. 1C). Of the few proteins enriched >2-fold, one candidate, CD3457, stood out because (i) its gene is strongly induced in the mother cell under the control of σ^E^ during sporulation, similar to *spoIVA* and *sipL* (30, 31), (ii) it was identified in a transposon mutagenesis screen for sporulation mutants (32), and (iii) it is conserved exclusively in the *Peptostreptococcaceae* family.

### CD3457 (SpoVQ) is a transmembrane protein that regulates heat-resistant spore formation.

**FIG 2:**
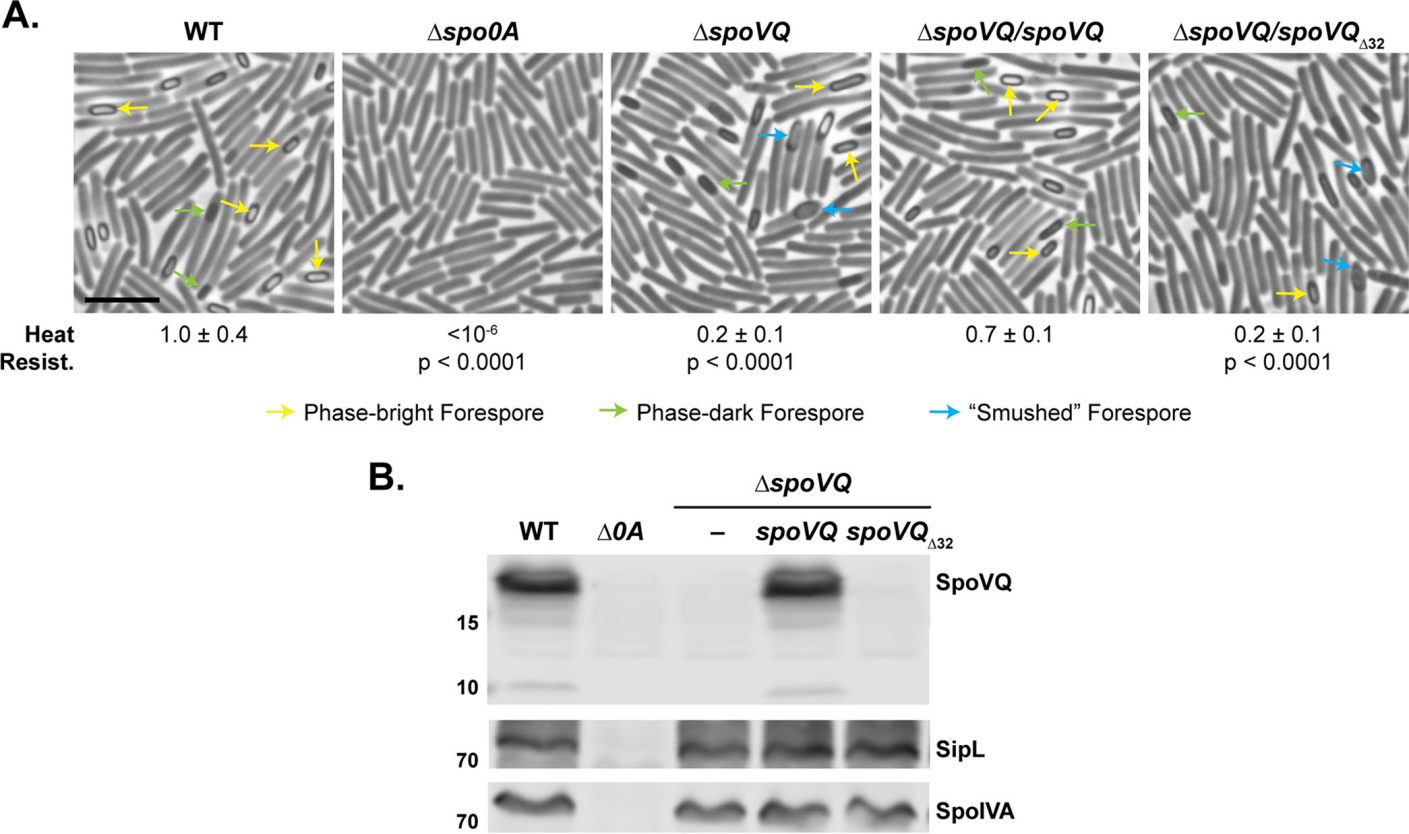
Loss of SpoVQ results in morphological and functional defects in spore formation. (A) Phase-contrast microscopy images of sporulating cultures of wild-type 630Δ*erm*-p (WT, *pyrE* restored) and the indicated strains ∼20 h after sporulation induction. Δ*spoVQ* was complemented with either wild-type *spoVQ* or *spoVQ*_Δ32_, the latter of which encodes an N-terminal truncation of SpoVQ’s transmembrane domain. Arrows mark mature phase-bright forepores (yellow), immature phase-dark forespores (green), and “smushed” forespores (blue), which appeared flattened relative to the oblong phase-dark or phase-bright forespores highlighted. Heat resistance efficiencies were calculated from 20- to 24-h sporulating cultures. The efficiencies represent the average ratio of heat-resistant spore CFU to total cells for a given strain relative to the WT based on a minimum of three biological replicates; the standard deviation is shown. The limit of detection of the assay is 10^−6^. Scale bar represents 5 μm. (B) Western blot analyses of strains shown in panel A using anti-SpoVQ, anti-SipL (20), and anti-SpoIVA antibodies.

To determine whether CD3457 (renamed SpoVQ for reasons detailed below) regulates coat formation and/or functional spore formation in C. difficile, we deleted *spoVQ* from C. difficile 630Δ*erm*Δ*pyrE* (29) and analyzed the sporulation phenotype of the resulting mutant using a heat resistance assay. This assay measures the capacity of sporulating cultures to produce spores that can survive a heat treatment that kills vegetative cells and germinate on media containing bile acid germinant (33). Loss of *spoVQ* resulted in a 5-fold reduction in heat-resistant spore formation (*P* < 0.0001). This defect was largely complemented by expressing a wild-type copy of *spoVQ* from the ectopic *pyrE* locus (Fig. 2A). Western blot analyses confirmed that the Δ*spoVQ*/*spoVQ* complementation strain produces SpoVQ at wild-type levels (Fig. 2B) and indicated that SpoIVA and SipL levels were unaffected by loss of SpoVQ.

Although SpoVQ is annotated as a conserved hypothetical protein, domain analyses predicted that SpoVQ carries an N-terminal transmembrane domain (Fig. S1A). To confirm that SpoVQ carries an N-terminal transmembrane domain, we compared the solubility of recombinant full-length SpoVQ relative to a variant lacking the N-terminal 32 residues in Escherichia coli. Consistent with the N-terminal 32 amino acids (aa) forming a transmembrane domain (SpoVQ_Δ32_), full-length SpoVQ was primarily observed in the insoluble fraction, whereas SpoVQ_Δ32_ was highly soluble (Fig. S1B).

10.1128/mSphere.00211-21.4 FIG S1 SpoVQ has an N-terminal transmembrane domain. (A) TMHMM prediction of transmembrane helices in SpoVQ. Topology prediction regarding whether the C-terminal soluble domain is secreted or cytosolic is ambiguous. (B) Ni^2+^-affinity purification of full-length His-tagged SpoVQ and His-tagged SpoVQ_Δ32_, which is missing its N-terminal 32 aa (predicted transmembrane domain). Whole-cell lysate (WCL), insoluble (IS), cleared lysate (soluble), flowthrough (FT), wash, and imidazole elution fractions from the purification were resolved by SDS-PAGE and Coomassie stained. Deletion of the transmembrane domain markedly increases the solubility of SpoVQ. Download FIG S1, TIF file, 0.3 MB. Copyright © 2021 Touchette et al. 2021 Touchette et al. https://creativecommons.org/licenses/by/4.0/ This content is distributed under the terms of the Creative Commons Attribution 4.0 International license.

To test whether the putative transmembrane domain is important for SpoVQ function, we complemented our Δ*spoVQ* strain with a construct that deletes the region encoding the transmembrane domain (*spoVQ*_Δ32_). This truncation construct failed to complement the heat resistance defect of Δ*spoVQ* (Fig. 2A). This functional defect likely results from the truncation destabilizing SpoVQ, since SpoVQ_Δ32_ was undetectable in Western blot analyses of sporulating cell lysates (Fig. 2B) even though the anti-SpoVQ antibody used was raised against SpoVQ_Δ32_.

To determine what stage of sporulation was being impacted by loss of SpoVQ, we analyzed the Δ*spoVQ* mutant strains by phase-contrast microscopy. Sporulating cultures of Δ*spoVQ* appeared to sporulate with similar frequency as wild type, but fewer mature phase-bright forespores were visible in Δ*spoVQ* relative to the wild type (Fig. 2A, yellow arrows). While mislocalized coat is easily observed by phase-contrast microscopy in Δ*spoIVA* and Δ*sipL* sporulating cells (20, 25, 34), mislocalized coat was not seen in Δ*spoVQ* sporulating cells. However, Δ*spoVQ* cells frequently produced phase-dark forespores with a “smushed” appearance, a phenotype we had not previously observed in C. difficile mutants (Fig. 2A, blue arrows). This smushed forespore phenotype was reversed in wild-type *spoVQ* but not the *spoVQ*_Δ32_ complementation strains, consistent with the heat resistance phenotypes measured.

### Cortex formation is impaired in Δ*spoVQ* spores.

**FIG 3:**
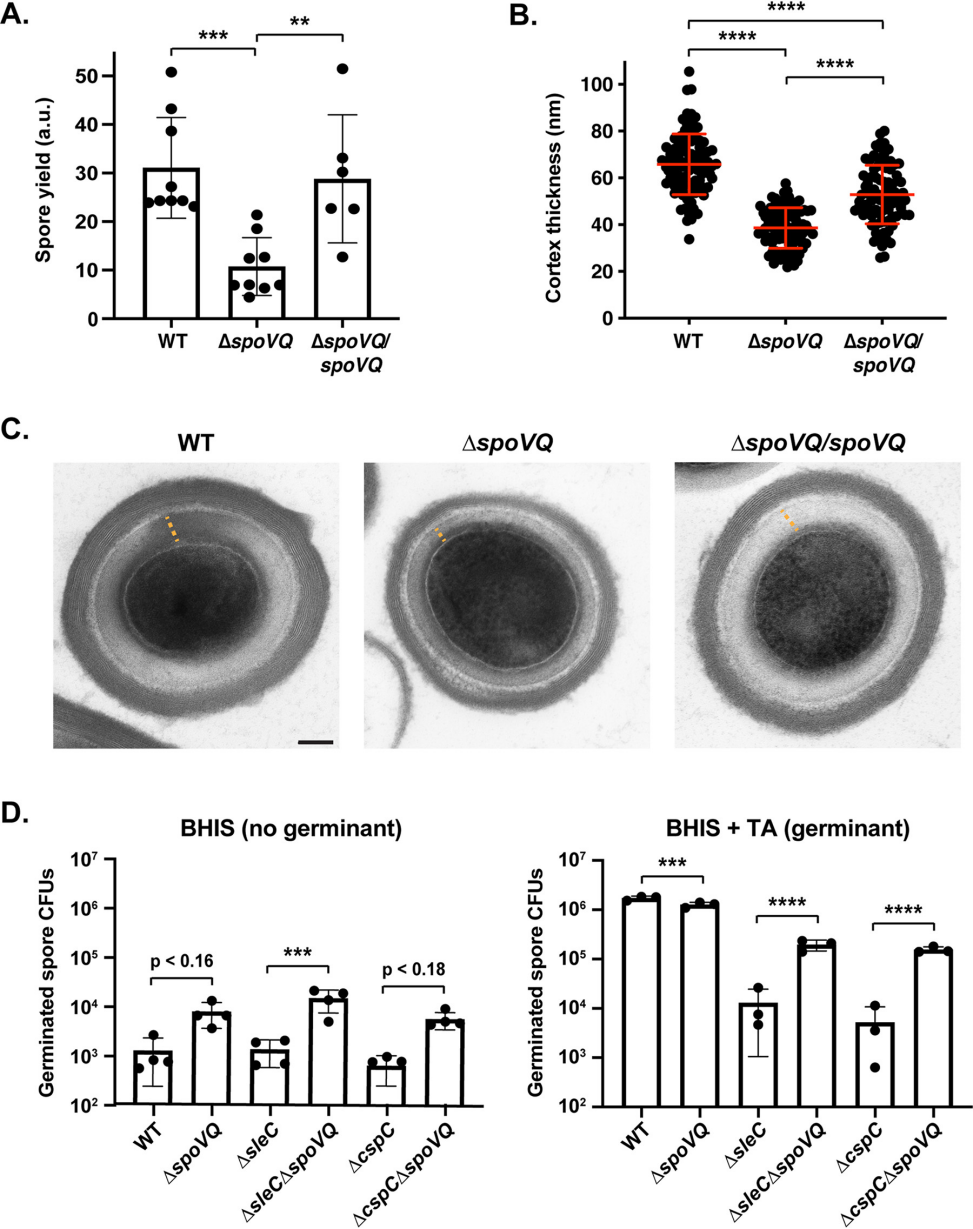
Δ*spoVQ* spores are purified less efficiently, produce a thinner cortex layer, and are prone to spontaneous germination. (A) Spore yields from purifications of WT, Δ*spoVQ*, and Δ*spoVQ* complementation strains from a minimum of six biological replicates. Yields were determined by measuring the optical density of spore purifications at 600 nm and are expressed in arbitrary units (a.u.). Statistical significance relative to the WT was determined using one-way ANOVA and Tukey’s test. ***, *P* < 0.0005; **, *P* < 0.01. (B) Cortex thickness (nm) of the indicated spores based on transmission electron microscopy (TEM) analyses. Measurements were made on a minimum of 85 spores per strain and are representative of two biological replicates. ****, *P* < 0.0001. (C) TEM of the indicated spores. The orange dashed line highlights the cortex thickness measured. Scale bar represents 100 nm. (D) Spontaneous germination of WT, Δ*spoVQ*, Δ*sleC*, and Δ*cspC* germination mutants and the indicated double mutant spores on rich (BHIS) medium lacking germinant (BHIS alone) and rich medium containing germinant (BHIS + taurocholate [TA]). Germination data are based on analyses of four independent spore preparations. Statistical significance relative to the parental strain is shown and derives from one-way ANOVA analyses and Tukey’s test. ****, *P* < 0.0001; ***, *P* < 0.005.

The smushed appearance of the spores led us to examine whether Δ*spoVQ* spores would be more difficult to purify. To this end, we compared the spore purification efficiencies of wild-type, Δ*spoVQ*, and Δ*spoVQ/spoVQ* strains. Loss of SpoVQ resulted in an ∼3-fold decrease in spore purification efficiency across three biological replicates (*P* < 0.0005; Fig. 3A), a decrease that was largely reversed in the Δ*spoVQ*/*spoVQ* complementation strain. The Δ*spoVQ* spores that survived the purification procedure were largely indistinguishable from wild-type spores when analyzed by phase-contrast microscopy (Fig. S2), although they were often less phase-bright (blue arrows, Fig. S2). Consistent with previous observations that spore refractivity correlates with cortex synthesis (12), *spoVQ* mutant spores in transmission electron microscopy (TEM) analyses produced cortex layers that were ∼60% the thickness of wild-type spores (66 ± 13 nm versus 38 ± 9 nm, *P* < 0.0001; Fig. 3B and C). The cortex layer of the Δ*spoVQ*/*spoVQ* complementation strain was ∼80% the thickness of wild-type spore (*P* < 0.0001; Fig. 3B), but the mean cortex thickness was still significantly higher than that of the parental Δ*spoVQ* mutant (*P* < 0.0001). Finally, the coat layers of Δ*spoVQ* spores were similar in thickness and appearance to wild-type spores, suggesting that loss of SpoVQ specifically affects cortex thickness (Fig. 3C).

10.1128/mSphere.00211-21.5 FIG S2 Δ*spoVQ* spore characterization. (A) Phase-contrast microscopy of purified wild-type (WT), Δ*spoVQ*, and Δ*spoVQ* complementation spores. Blue arrows highlight spores that are slightly darker than other spores. (B) Western blot analyses of Δ*spoVQ*, Δ*cotL*, and germination mutant spores. CotA, SpoIVA, and SipL are coat proteins (20, 35), while SleC, CspC, and CspB are all predicted to be cortex-localized (10, 63). C. perfringens SleC was previously localized to the cortex region using immunogold labeling (64). Western blots are representative of three biological replicates. Download FIG S2, TIF file, 0.8 MB. Copyright © 2021 Touchette et al. 2021 Touchette et al. https://creativecommons.org/licenses/by/4.0/ This content is distributed under the terms of the Creative Commons Attribution 4.0 International license.

Interestingly, the thinner cortex of Δ*spoVQ* spores resembled that of *cotL* mutant spores (26). Like SpoVQ, CotL is a mother cell-specific, σ^E^-regulated protein found exclusively in the *Peptostreptococcaceae* family that affects cortex thickness (26). Loss of CotL also causes coat encasement defects and reduces incorporation of proteins predicted to be part of the cortex layer. To compare the *spoVQ* and *cotL* mutant phenotypes more directly, we analyzed the levels of coat and putative cortex proteins in *spoVQ* and *cotL* mutant spores. These Western blot analyses indicated that Δ*spoVQ* spores contain wild-type levels of outer coat proteins such as CotA (35) and putative cortex-localized proteins such as the SleC cortex lytic enzyme and germinant signaling proteins, CspC and CspB (10) (Fig. S2B). In contrast, Δ*cotL* spores contained dramatically reduced levels of these proteins (and of the basement layer proteins, SpoIVA and SipL [25]), similar to prior analyses of a *cotL* TargeTron mutant (26). Thus, while the cortex thickness phenotype of Δ*spoVQ* spores resembled those of Δ*cotL* spores, these two mutant strains exhibit notable differences in their protein composition (Fig. S2B), suggesting that they impact C. difficile spore formation using different mechanisms.

### *spoVQ* mutant spores are prone to spontaneous germination.

The thinner cortex layer of *ΔspoVQ* spores prompted us to consider whether these mutant spores would be more likely to prematurely germinate. This question was prompted by the smushed morphology of Δ*spoVQ* forespores, which somewhat resembled the phase-dark appearance of Bacillus subtilis mutant spores that prematurely germinate due to defects in spore assembly (36). In these B. subtilis mutants, deletion of germinant receptor genes partially rescues their heat-resistant spore formation defects, many of which are due to impaired cortex formation. To test whether Δ*spoVQ* forespores with a smushed appearance were prematurely germinating, we assessed whether loss of key germinant signaling proteins would prevent premature germination in Δ*spoVQ* spores. For these analyses, we deleted *cspC*, which encodes the likely bile acid germinant receptor (37, 38), or *sleC*, which encodes the cortex lytic enzyme that degrades the protective cortex layer during germination (39, 40), from the ΔspoVQ strain. Both these proteins are critical for bile acid germinant signal transduction and thus C. difficile spore germination (37, 39, 40). Contrary to our hypothesis, deletion of *sleC* or *cspC* did not improve the purification efficiency of Δ*spoVQ* spores (data not shown). In fact, the Δ*spoVQ*Δ*sleC* and Δ*spoVQ*Δ*cspC* double mutants germinated 15- to 30-fold, respectively, more on rich media containing germinant than single mutants lacking these critical germination proteins (Fig. 3D). However, this enhanced germination was not statistically significant relative to the wild type.

Notably, while Δ*spoVQ* spores germinated at close to wild-type levels on rich media containing germinant (1.4-fold reduction, *P* < 0.001), when the mutant spores were plated on rich media lacking germinant, they germinated at ∼6-fold higher levels than wild-type spores (*P* ∼ 0.17). Furthermore, deletion of *spoVQ* from either Δ*sleC* or Δ*cspC* spores enhanced this spontaneous germination in the absence of germinant by 14- and 10-fold, respectively (Fig. 3D); the former result was statistically significant (*P* < 0.0005); the latter was not. Since spontaneous germination has been defined as spore germination that occurs in the absence of (i) germinant and/or (ii) key germinant signaling proteins such as germinant receptors (e.g., CspC) or cortex lytic enzymes (e.g., SleC) (41–43), our results indicate that loss of SpoVQ promotes spontaneous germination of wild-type and germinant signaling mutant spores, a phenotype that could be linked to the thinner cortex of Δ*spoVQ* spores (Fig. 3B).

### SpoVQ is required for cortex formation in the absence of SpoIVA or SipL.

Since SpoVQ affects cortex synthesis (Fig. 3) and binds SipL and/or SpoIVA (Fig. 1), which themselves impact cortex thickness (23, 25), we wondered whether SpoVQ was required for cortex synthesis in Δ*spoIVA* or Δ*sipL* strains. To test this possibility, we deleted *spoVQ* from either Δ*spoIVA* or Δ*sipL* strains and analyzed the resulting double mutants morphologically using phase-contrast microscopy and TEM analyses. While developing forespores with phase-dark outlines were observed in wild-type (WT), Δ*spoIVA*, Δ*sipL*, and Δ*spoVQ* strains (orange arrows), these dark outlines were not observed in the double mutant Δ*spoIVA*Δ*spoVQ* and Δ*sipL*Δ*spoVQ* strains (Fig. S3A). Phase-dark outlines around forespores are typically observed when forespores become phase-bright due to the dehydration of the forespore cytosol as it matures (12), which occurs when the thick cortex layer is synthesized. To assess whether Δ*spoIVA*Δ*spoVQ* and Δ*sipL*Δ*spoVQ* strains produced a cortex layer, we analyzed sporulating cultures of these cells using TEM.

10.1128/mSphere.00211-21.6 FIG S3 Morphological analyses of sporulating cultures of Δ*spoVQ*, Δ*sipL*, and Δ*spoIVA* single and double mutants. (A) Phase-contrast microscopy analyses. Phase-bright forespores (yellow arrows) are detected in wild-type and Δ*spoVQ* spores but not in Δ*sipL* or Δ*spoIVA* strain backgrounds. “Smushed” forespores (blue arrows) are visible in strains lacking *spoVQ*, while mislocalized coat (pink arrows) is visible in strains lacking either *sipL* or *spoIVA*. Δ*sipL* and Δ*spoIVA* strains occasionally make forespores with a distinct phase-dark outline (orange arrows) that likely represents synthesized cortex. (B and C) Transmission electron microscopy analyses of sporulating culture shown in panel A. Examples of the phenotypes scored for cortex thickness (B) and forespore color/texture (C) are shown. Thin yellow lines in panel B highlight the cortex region where detectable. Scale bars represent 500 nm. Download FIG S3, TIF file, 2.6 MB. Copyright © 2021 Touchette et al. 2021 Touchette et al. https://creativecommons.org/licenses/by/4.0/ This content is distributed under the terms of the Creative Commons Attribution 4.0 International license.

**FIG 4:**
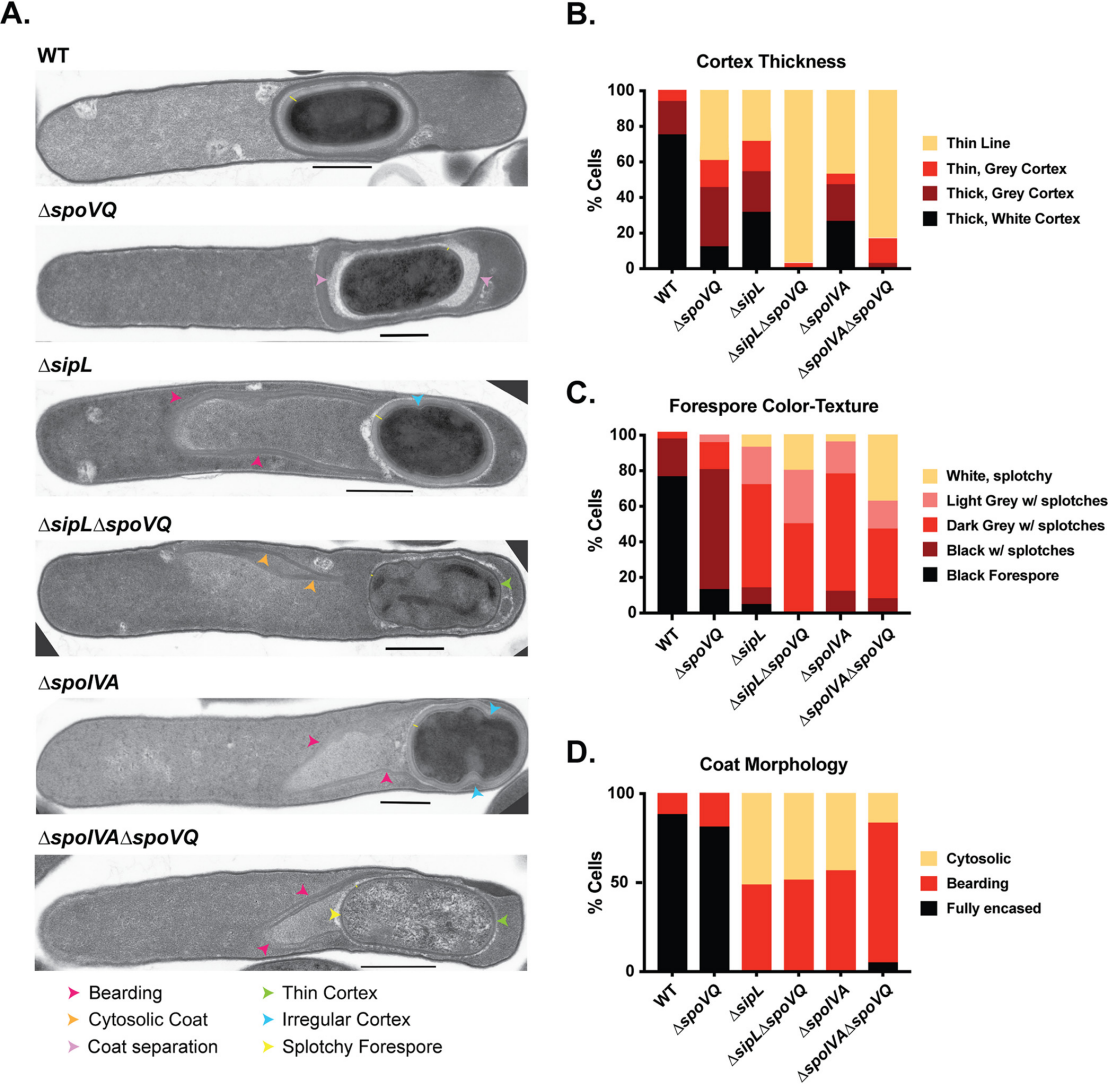
SpoVQ regulates cortex formation in a SpoIVA- and SipL-dependent manner. (A) Transmission electron microscopy (TEM) of wild-type (WT), Δ*spoVQ*, Δ*sipL*, Δ*spoIVA*, and the indicated double mutants 23 h after sporulation induction. Arrows highlight various phenotypes observed in the different mutant strains. Δ*spoVQ* sporulating cells exhibited many of these phenotypes. (B) Cortex thickness and electron density in the indicated strains. Mature cortex appears as an electron-light layer on top of a darker germ cell wall. Cortex designated “thin line” likely represents the darker germ cell wall of the forespore, a phenotype that was particularly prominent in Δ*spoIVA*Δ*spoVQ* and Δ*sipL*Δ*spoVQ* mutants (green arrow in panel A). (C) Electron density of forespores in the indicated strains as described by color and texture. Electron-dark, uniform forespores likely represent mature forespores whose core (cytosol) has partially dehydrated. Splotches likely arise during TEM sample processing and may be more likely in forespores that are more hydrated. (D) Coat morphology of the indicated strains. “Fully encased” represents polymerized coat encircling the forespore. “Bearding” refers to polymerized coat sloughing off the forespore (pink arrows in panel A). “Cytosolic” refers to polymerized coat mislocalizing entirely from the forespore into the cytosol (orange arrows in panel A). “Coat separation” refers to coat that appears to form a balloon around the forespore, i.e., is not closely associated with the entire forespore.

In developing forespores, mature cortex appears thick and electron-light (white) in the micrographs due to the large amount of cortex peptidoglycan synthesized during sporulation and its reduced level of cross-linking (12). In contrast, immature cortex layers are thinner and darker (dark gray) in electron micrographs. Wild-type forespores primarily produced a thick cortex layer that was electron-light (white), i.e., fully mature (Fig. 4A and B). A thick, mature cortex layer was also observed in ∼30% of Δ*spoIVA* and Δ*sipL* forespores, consistent with our prior report that SpoIVA and SipL are not essential for cortex formation in C. difficile (20). Nevertheless, as described earlier, Δ*spoIVA* and Δ*sipL* forespores exhibit cortex abnormalities, with the majority of Δ*spoIVA* and Δ*sipL* sporulating cells making cortex that is thinner and/or darker than that of the wild type (Fig. 4A and Fig. S3B). The cortex in Δ*spoIVA* and Δ*sipL* sporulating cells frequently exhibited indentations or areas of abnormal thickness as previously described (18, 23, 25). In contrast, Δ*spoVQ* sporulating cells rarely produced a thick, mature cortex layer (∼10%, green arrows, Fig. 4B and Fig. S3B); instead, this layer was markedly darker and thinner (Fig. 4A). Notably, the cortex layer was even thinner in Δ*spoIVA*Δ*spoVQ* and Δ*sipL*Δ*spoVQ* sporulating cells, with the majority of cells in the double mutants producing a dark, thin cortex layer that was difficult to distinguish from the germ cell wall. The double mutants, like the parental mutants Δ*sipL* and Δ*spoIVA*, frequently produced forespores with abnormal shapes. Taken together, these results strongly suggest that formation of the cortex layer in Δ*spoIVA* and Δ*sipL* mutants depends on the presence of SpoVQ, further highlighting the importance of this hypothetical protein in regulating cortex formation.

The reduced thickness and higher electron density of the cortex layer in Δ*spoVQ* spores correlated with lighter electron density in the forespore cytosol (also known as the core; Fig. 4C and Fig. S3C). Less than 20% of Δ*spoVQ* spores produced an electron-dense (dark) forespore, which could suggest that Δ*spoVQ* spores may be more hydrated than wild-type spores. The forespores of Δ*spoIVA* and Δ*sipL* derivatives were also less electron dense and thus inversely correlated with the thickness of their cortex layers.

Despite these differences in forespore core and cortex appearance in Δ*spoVQ* cells, coat layers appeared to encase Δ*spoVQ* forespores (Fig. 4D), a finding that is consistent with our Western blot studies of Δ*spoVQ* spores (Fig. S2). As reported previously, loss of either SpoIVA or SipL largely abrogated coat encasement of the forespore irrespective of whether SpoVQ was present. In Δ*sipL* and Δ*spoIVA* mutants, polymerized coat was observed sloughing off the forespore (termed “bearding”) or completely mislocalized to the cytosol (Fig. 4D). Thus, despite SpoVQ’s effect on cortex thickness, it does not appear to affect coat encasement, again in contrast with CotL’s effect on both coat encasement and cortex formation in C. difficile (26).

### SpoVQ specifically localizes to forespore membranes in a SpoIVA- and SipL-independent manner, although SpoVQ affects SipL localization to the forespore.

**FIG 5:**
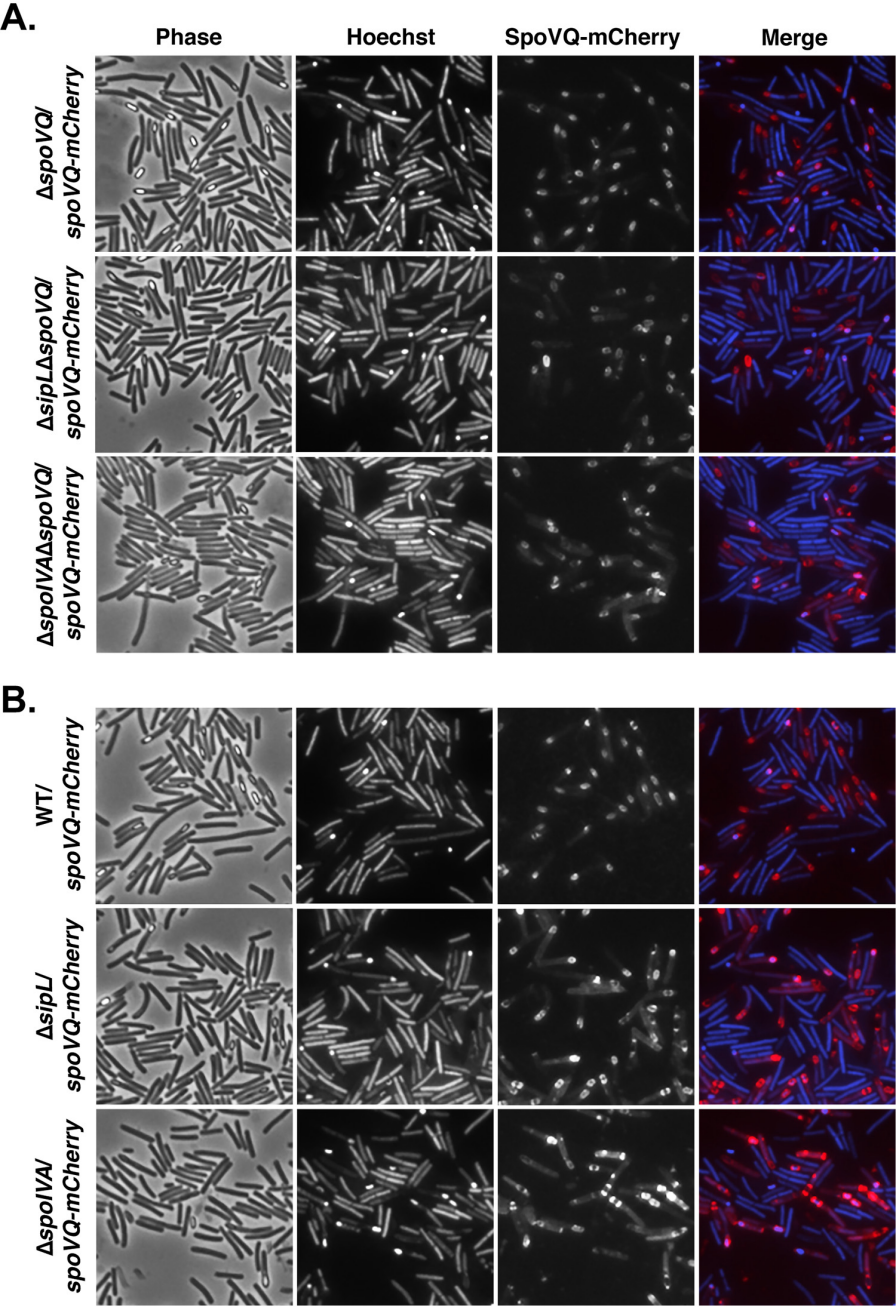
SpoVQ localizes around the forespore in a largely SpoIVA- and SipL-independent manner. (A) SpoVQ-mCherry localization in Δ*spoVQ*, Δ*sipL*Δ*spoVQ*, and Δ*spoIVA*Δ*spoVQ* strains, where the fusion protein is the only version of SpoVQ present. (B) SpoVQ-mCherry localization in wild-type, Δ*sipL*, and Δ*spoIVA* strains, where the fusion protein and untagged SpoVQ are both produced. A slight increase in cytosolic signal in SpoVQ-mCherry was observed in the Δ*spoIVA* strain background. Sporulating cells were visualized by phase-contrast microscopy (phase); the nucleoid was visualized with Hoechst. In the “merge” image, SpoVQ-mCherry fluorescence is shown in red and Hoechst staining is shown in blue. Images are representative of the results of three biological replicates.

Our finding that SpoVQ regulates cortex synthesis in a SpoIVA- and SipL-dependent manner prompted us to consider whether SpoVQ affected SpoIVA and/or SipL localization around the forespore and vice versa. To address these questions, we analyzed the localization dependencies of SpoVQ, SpoIVA, and SipL using fluorescent protein fusions. We constructed a C-terminal mCherry fusion to SpoVQ and analyzed its localization around the forespore in merodiploid and Δ*spoVQ* backgrounds. In both these strain backgrounds, SpoVQ-mCherry specifically localized around the forespore (Fig. 5). This observation suggests that even though SpoVQ-mCherry should have the capacity to localize to all mother cell-derived membranes because it is produced under the control of σ^E^ (30, 31), it concentrates within the mother cell-derived forespore membrane through an unknown mechanism. Importantly, the SpoVQ-mCherry fusion fully complemented Δ*spoVQ* (data not shown) and primarily produced a full-length fluorescent protein fusion (Fig. S4).

10.1128/mSphere.00211-21.7 FIG S4 Western blot analyses of tagged *spoVQ* strains. *spoVQ-FLAG_3_* and *spoVQ-mCherry* encode C-terminal epitope and fluorescent protein fusions to SpoVQ, respectively. The asterisk represents a cleavage product of SpoVQ-mCherry. Some free mCherry is detected. The antibodies used for each blot are shown below. The result is representative of at least two biological replicates. Download FIG S4, TIF file, 0.3 MB. Copyright © 2021 Touchette et al. 2021 Touchette et al. https://creativecommons.org/licenses/by/4.0/ This content is distributed under the terms of the Creative Commons Attribution 4.0 International license.

Since our biochemical (Fig. 1) and genetic data (Fig. 4) suggested an interaction between SpoVQ and the SipL-SpoIVA complex, we assessed whether SpoIVA or SipL affects SpoVQ’s localization around the forespore. To this end, we complemented our Δ*spoIVA*, Δ*sipL*, Δ*spoIVA*Δ*spoVQ*, and Δ*sipL*Δ*spoVQ* double mutant strains with *spoVQ-mCherry* and analyzed the localization of this protein fusion during sporulation. SpoVQ-mCherry primarily localized around the forespore in the absence of either SpoIVA or SipL (Fig. 5), regardless of whether untagged SpoVQ was also made. However, loss of SpoIVA seemed to increase the cytosolic SpoVQ-mCherry signal. These results suggest that SpoIVA may promote SpoVQ localization around the forespore, but neither SpoIVA nor SipL is absolutely essential for SpoVQ to concentrate in the mother cell-derived outer forespore membrane.

**FIG 6:**
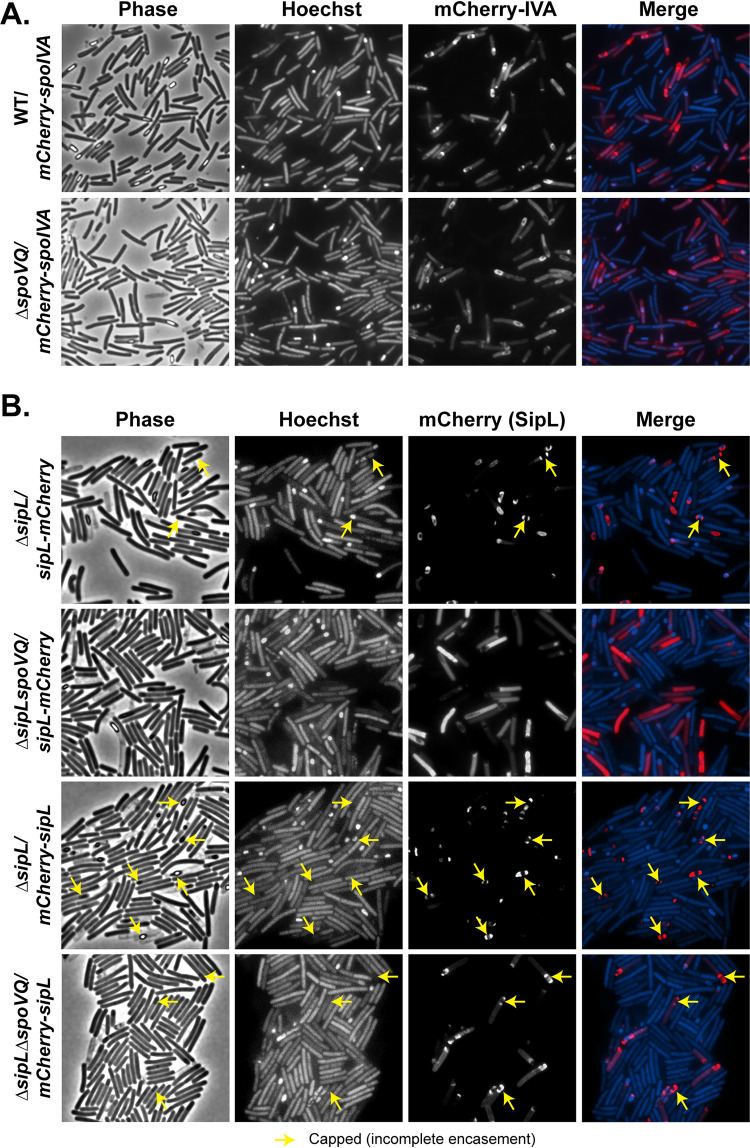
SpoVQ affects the localization of SipL-mCherry but not mCherry-SipL or mCherry-SpoIVA fusions. (A) mCherry-SpoIVA localization in wild-type versus Δ*spoVQ* strains, where mCherry-SpoIVA and untagged SpoIVA are both produced in these merodiploid strains. (B) SipL-mCherry or mCherry-SipL (C-terminal versus N-terminal mCherry fusion, respectively) localization in Δ*sipL* versus Δ*sipL*Δ*spoVQ* strain backgrounds. In these strains, the fusion protein is the only version of SipL present. The *mCherry-sipL* fusion construct complements Δ*sipL* less efficiently than *sipL-mCherry* in terms of heat-resistant spore formation (Fig. S5), consistent with mCherry-SipL encasing the forespore less efficiently than SipL-mCherry (yellow arrows, “capped” distribution). Cytosolic mCherry signal was elevated in the absence of SpoVQ, particularly with the C-terminal SipL-mCherry fusion. Sporulating cells were visualized by phase-contrast microscopy (phase); the nucleoid was visualized with Hoechst. In the “merge” image, mCherry fluorescence of the indicated fusion proteins is shown in red and Hoechst staining is shown in blue. Images are representative of the results of three biological replicates.

To determine whether SpoVQ affects SpoIVA and/or SipL localization around the forespore, we analyzed the localization of previously published mCherry-SpoIVA and SipL-mCherry fusions in the presence and absence of SpoVQ. To localize mCherry-SpoIVA, we introduced a construct encoding an mCherry-SpoIVA fusion protein (25) into the *pyrE* locus of the Δ*spoVQ* mutant. It was necessary to generate this *spoIVA* merodiploid strain because untagged SpoIVA promotes encasement of the partially functional mCherry-SpoIVA fusion (18). SpoVQ did not appear to affect localization of mCherry-SpoIVA around the forespore (Fig. 6A), indicating that SpoIVA forespore encasement does not depend on SpoVQ. In contrast, localization of a functional SipL-mCherry fusion protein was significantly impaired in the absence of SpoVQ, with much of the SipL-mCherry signal redistributing to the cytosol of Δ*sipL*Δ*spoVQ*/*sipL-mCherry* cells (Fig. 6B). In this strain, SipL-mCherry is the only copy of SipL, since we previously showed that coproduction of the fusion with untagged SipL increased the cytosolic signal of SipL-mCherry (25). Western blot analyses indicated that SipL-mCherry levels were elevated in the Δ*spoVQ* background relative to the wild type and the Δ*sipL* complementation strain (Fig. S5). The increased mCherry signal in the cytosol of Δ*sipL*Δ*spoVQ*/*sipL-mCherry* could be due to increased liberation of mCherry from the higher levels of SipL-mCherry. Despite this degradation, some SipL-mCherry fusion was observed around the forespore (Fig. 6B, yellow arrows), although the functionality of the fusion was reduced ∼10-fold relative to Δ*spoVQ* alone (Fig. S5). Indeed, ∼100-fold fewer heat-resistant spores were detected in Δ*sipL*Δ*spoVQ*/*sipL-mCherry* relative to Δ*sipL*/*sipL-mCherry* (Fig. S5).

10.1128/mSphere.00211-21.8 FIG S5 Western blot analyses of strains carrying mCherry-fusions to SipL and SpoIVA. *mCherry-spoIVA* (*IVA*) was conjugated into a wild-type and Δ*spoVQ* strain background to create merodiploid strains. *sipL-mCherry* (25) and *mCherry-sipL* constructs were conjugated into a Δ*sipL* strain so that the fusion protein is the only SipL version present. Functionality of the fusion proteins was assessed using a heat resistance assay (heat res. [33]). The results are based on a minimum of three biological replicates (with the exception of Δ*sipLΔspoVQ*/*mCherry-sipL*, which is based on two biological replicates). (Δ*sipL/sipL-mCherry* versus Δ*sipLΔspoVQ/sipL-mCherry*, *P* < 0.005; Δ*sipL/sipL-mCherry* versus Δ*sipLΔspoVQ/mCherry-sipL*, *P* < 0.005). Minimal degradation of fluorescent protein fusions was observed. It should also be noted that SipL-mCherry and mCherry-SipL are virtually identical in size. Results are representative of two biological replicates. Download FIG S5, TIF file, 0.3 MB. Copyright © 2021 Touchette et al. 2021 Touchette et al. https://creativecommons.org/licenses/by/4.0/ This content is distributed under the terms of the Creative Commons Attribution 4.0 International license.

Given that loss of SpoVQ caused much of the SipL-mCherry signal to mislocalize to the cytosol and reduced the function of this fusion protein by ∼10-fold (Fig. S5), we wondered whether the fusion of mCherry to the C terminus of SipL was interfering with SipL function specifically in the absence of SpoVQ. SipL’s C-terminal LysM domain binds directly to SpoIVA (20, 25) and may also bind peptidoglycan (25, 44, 45), so fusions to SipL’s C terminus could disrupt binding to SpoVQ, if SpoVQ is oriented into the intermembrane space. Consistent with this hypothesis, moving the mCherry fusion to the N terminus of SipL increased localization of mCherry-SipL to the forespore even in the absence of SpoVQ (Fig. 6B). In particular, the majority of the mCherry signal in Δ*sipL*Δ*spoVQ*/*mCherry-SipL* localized to the forespore, in contrast to Δ*sipL*Δ*spoVQ*/*sipL-mCherry*. Nevertheless, loss of SpoVQ increased the cytosolic mCherry-SipL signal in Δ*sipL*Δ*spoVQ* relative to the wild-type *sipL* complementation strain.

Surprisingly, the function of the N-terminal mCherry-SipL was reduced even further than the C-terminal fusion in the absence of SpoVQ (∼10-fold fewer heat-resistant spores were observed in Δ*sipL*Δ*spoVQ*/*mCherry-sipL* relative to Δ*sipL*Δ*spoVQ*/*sipL-mCherry*; Fig. S5). The reduced function of mCherry-SipL may simply reflect the reduced levels of this fusion protein relative to SipL-mCherry or untagged SipL (Fig. S5). This reduction in mCherry-SipL may explain why the mCherry-SipL fusion (i.e., Δ*sipL*/*mCherry-sipL*) encased the forespore less efficiently than SipL-mCherry (Fig. 6B). The Δ*sipL*/*mCherry-sipL* forespores also appeared more round than wild-type and Δ*sipL*/*sipL-mCherry* forespores, consistent with the ∼3-fold reduction in heat-resistant spores in Δ*sipL*/*mCherry-sipL* relative to WT (Fig. S5). Taken together, these results indicate that a C-terminal fusion of mCherry to SipL disrupts SipL-mCherry’s encasement of the forespore, specifically in the absence of SpoVQ.

### Recombinant SpoVQ directly binds both SpoIVA and SipL.

The localization (Fig. 5 and 6) and double mutant analyses (Fig. 4) supported a genetic interaction between SpoVQ and both SipL and SpoIVA, while the coimmunoprecipitation analyses revealed a biochemical interaction between SpoVQ, SipL, and SpoIVA during C. difficile sporulation (Fig. 1). To test whether SpoVQ directly binds SipL and/or SpoIVA, we assessed whether immunoprecipitating FLAG-tagged SpoVQ would pull down untagged SipL and/or SpoIVA in C. difficile sporulating cell lysates. For these analyses, we complemented Δ*spoVQ* with a strain producing C-terminally FLAG-tagged SpoVQ and coimmunoprecipitated SpoVQ-FLAG_3_ in the presence of detergent because SpoVQ is a bitopic membrane protein. Immunoprecipitation of FLAG-tagged SpoVQ failed to pull down SipL, although small amounts of SpoIVA coimmunoprecipitated with SpoVQ-FLAG_3_, implying that SpoVQ may bind SpoIVA albeit weakly in the presence of NP-40 detergent (Fig. S6).

10.1128/mSphere.00211-21.9 FIG S6 Coimmunoprecipitation analyses of FLAG-tagged *sipL* and *spoVQ* from C. difficile sporulating cell lysates. (A) FLAG-tagged SipL and SpoVQ were immunoprecipitated from cleared lysates prepared from the indicated C. difficile Δ*sipL* and Δ*spoVQ* complementation strains, respectively, using anti-FLAG magnetic beads. No detergent was present in these analyses. (I, input fraction) After several washes, proteins retained by the beads were eluted using FLAG peptide (E, elution fraction). The untagged *sipL* and *spoVQ* complementation strains serve as negative controls to assess specific binding of FLAG-tagged proteins to untagged beads. Sporulation was induced for 24 h before lysates were prepared. Untagged SpoIVA was pulled down robustly by SipL-FLAG_3_, while untagged SpoVQ copurified with SipL-FLAG_3_ with lower apparent efficiency. The immunoprecipitations shown are representative of three independent biological replicates. (B) SipL-FLAG_3_ and SpoVQ-FLAG_3_ coimmunoprecipitations were performed in the presence of 0.1% NP40 detergent and analyzed similarly to panel A. Lower levels of untagged SpoIVA were copurified with SipL-FLAG_3_ and SpoVQ-FLAG_3_, respectively. Download FIG S6, TIF file, 1.1 MB. Copyright © 2021 Touchette et al. 2021 Touchette et al. https://creativecommons.org/licenses/by/4.0/ This content is distributed under the terms of the Creative Commons Attribution 4.0 International license.

Since our original coimmunoprecipitation analyses using FLAG-tagged SipL did not include detergent because SipL and SpoIVA are soluble proteins, we tested whether the inclusion of detergent would affect the efficiency of SpoVQ binding to SipL-FLAG_3_. While FLAG-tagged SipL pulled down untagged SpoIVA (albeit at reduced levels) when NP-40 detergent was included, no SpoVQ was detected in the SipL-FLAG_3_ pulldown (Fig. S6). In contrast, when detergent was not included in these analyses, trace amounts of SpoVQ were detected in the SipL-FLAG_3_ pulldowns (Fig. S6). These findings suggest that the interaction between SipL and SpoVQ is either unstable in the presence of detergent or nonspecific in the absence of detergent.

Despite these negative results, the genetic interactions we observed between SpoVQ, SpoIVA, and SipL (Fig. 4) prompted us to use an alternative method to assess binding between SpoVQ and SipL and/or SpoIVA. Specifically, we used a recombinant coaffinity purification strategy that can detect binding between SpoIVA and SipL (20). This heterogeneous expression strategy allows proteins to be produced at higher levels and more readily measures direct binding. For these experiments, we coproduced His-tagged SpoVQ_Δ32_ (soluble domain) with either SipL or SpoIVA in E. coli and tested whether SipL or SpoIVA, respectively, would be present in the SpoVQ_Δ32_-His_6_ pulldowns. As a negative control, we used the soluble cysteine protease domain (CPD) derived from Vibrio cholerae MARTX toxin (46, 47). When His-tagged SpoVQ_Δ32_ was affinity-purified in the presence of either untagged SipL or SpoIVA, both proteins were enriched in the SpoVQ_Δ32_-His_6_ pulldowns. In contrast, when His-tagged SpoVQ_Δ32_ was affinity-purified in the presence of untagged CPD, no enrichment of the CPD was observed. These results indicate that recombinant SipL and SpoIVA can both interact with the soluble domain of SpoVQ at least in E. coli lysates.

## DISCUSSION

Coat formation in Clostridioides difficile exhibits notable differences relative to Bacillus subtilis (10). For example, SipL is a clostridial-specific coat protein essential for coat localization to the forespore (20). Recent work has also identified *Peptostreptococcaceae* family-specific spore morphogenetic proteins that regulate C. difficile coat assembly in addition to other processes. CotL impacts C. difficile coat and cortex assembly (26), and CdeC is required for proper coat and exosporium assembly (48). These findings strongly suggest that the *Peptostreptococcaceae* family uses distinct pathways to assemble infectious spores (10).

**FIG 7:**
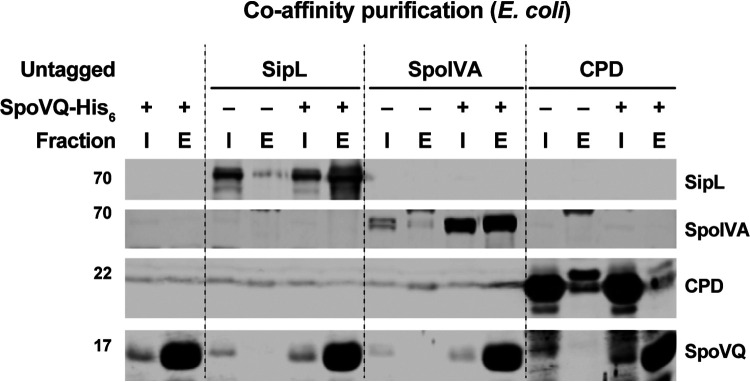
SpoVQ can separately bind SpoIVA and SipL in coaffinity purification analyses. Coaffinity purification analyses of His-tagged SpoVQ_Δ32_, which lacks its N-terminal transmembrane domain, with SipL, SpoIVA, and the CPD, a cysteine protease domain from Vibrio cholerae (46). The indicated proteins were coproduced in E. coli, and cleared, soluble lysates were prepared. SpoVQ_Δ32_-His_6_ was affinity-purified using Ni-NTA resin, and high imidazole was used to elute SpoVQ_Δ32_-His_6_ and any associated proteins. Cleared lysate (input, I) and elution (E) samples were analyzed by Western blotting using anti-SipL, anti-SpoIVA, anti-CPD, and anti-SpoVQ antibodies.

In this study, we have identified SpoVQ as yet another *Peptostreptococcaceae* family-specific spore morphogenetic protein. Our analyses indicate that SpoVQ (CD3457) is a mother cell-specific bitopic membrane protein that specifically localizes to the outer forespore membrane (Fig. 5) and regulates cortex thickness. The thinner cortex of Δ*spoVQ* spore regulation likely promotes higher levels of spontaneous germination (Fig. 3). SpoVQ is also essential for cortex synthesis in *spoIVA* and *sipL* mutants (Fig. 4), which already exhibit abnormalities in cortex thickness and electron density (Fig. 4 [23, 25]). Since SpoVQ directly binds both SpoIVA and SipL (Fig. 7) and also influences SipL-mCherry localization to the forespore (Fig. 6), our data collectively suggest that SpoVQ functions to link coat and cortex assembly in C. difficile.

A functional link between coat and cortex formation has long been established in B. subtilis, since *spoIVA* and *spoVM* mutants fail to make cortex and exhibit coat localization defects (16, 17, 21, 49). Recent work has shed light on the mechanism linking these two critical processes with the identification of SsdC as a Bacilli-specific, mother cell-derived membrane protein. SsdC localizes to the outer forespore membrane, regulates cortex synthesis, and genetically interacts with several basement layer coat morphogenetic proteins (50). While B. subtilis SsdC and C. difficile SpoVQ do not share sequence homology, they may share similar functions in linking cortex and coat formation through direct protein-protein interactions.

Addressing how SpoVQ impacts cortex formation in C. difficile is challenging given that the mechanisms controlling cortex synthesis in C. difficile remain poorly defined. Three of the enzymes critical for cortex peptidoglycan synthesis in B. subtilis are conserved in C. difficile, namely, the SEDS glycosyltransferase-class B penicillin-binding protein transpeptidase pair, SpoVE and SpoVD, and the SpoVB flippase (11, 12, 15, 51), but SpoVD is the only C. difficile protein that has been shown to regulate cortex synthesis to date (52). Analyzing the localization dependence of SpoVD and the remaining enzymes on C. difficile SpoVQ would address whether SpoVQ alters cortex thickness by impacting the localization and/or activity of these peptidoglycan (PG) synthesizing enzymes. Studies directed at identifying binding partners of SpoVQ (beyond SpoIVA and SipL) using our functional epitope-tagged construct (Fig. S6) may also provide insight into how SpoVQ modulates cortex synthesis.

Ironically, while we originally identified SpoVQ as a binding partner of SipL via coimmunoprecipitation (Fig. 1), this interaction was not stable in the presence of detergent. Validating this interaction in the presence of detergent was necessary to rule out the possibility that SpoVQ indirectly binds SipL in the context of membrane micelles containing direct SipL interactors. Given that the stability of SpoIVA and SipL binding decreased markedly in the presence of detergent (Fig. S6), it is possible that SipL and SpoVQ specifically bind during C. difficile spore formation but that the interaction is destabilized by detergent (Fig. 1 and Fig. S6). Future work should more rigorously assess direct binding between these proteins during C. difficile sporulation, potentially by using cross-linking followed by coimmunoprecipitation analyses (53) or using colocalization analyses (54). Identifying the regions within recombinant SpoIVA and SipL that bind SpoVQ’s soluble domain and whether SpoVQ binds the SpoIVA-SipL complex more efficiently would also provide important insight into this interaction.

Another important question is whether SpoVQ’s soluble domain is oriented toward the intermembrane space or within the mother cell cytosol. TMHMM analyses predict relatively similar probabilities that SpoVQ’s C-terminal soluble domain is secreted versus remaining cytosolic (Fig. S1A). Given the impact that SpoVQ has on cortex synthesis (Fig. 3), it seems more likely that the C-terminal domain is secreted into the intermembrane space, where it could regulate cortex synthesis. If this is the case, it raises the question of how SpoVQ, SpoIVA, and SipL physically interact, since SpoIVA and SipL are made in the mother cell cytosol. Recent work in B. subtilis suggests that some coat proteins can bridge the outer forespore membrane and bind cortex peptidoglycan. The B. subtilis coat morphogenetic protein, SafA, binds peptidoglycan (45) and the coat morphogenetic protein, SpoVID (55). Thus, SafA has a mechanism to reach across the outer forespore membrane despite lacking a predicted transmembrane domain. SafA binding to peptidoglycan depends on its N-terminal LysM domain (45), which is also required for SafA to bind SpoVID (55). Like B. subtilis SafA, C. difficile SipL carries a LysM domain that directly binds SpoIVA (20, 25), but whether SipL’s LysM domain binds peptidoglycan remains to be tested. Interestingly, SipL’s LysM domain is C-terminal, and loss of SpoVQ reduces the function of a C-terminal mCherry fusion to SipL (Fig. 6). Given that a C-terminal, but not N-terminal, fusion of mCherry to SipL disrupted SipL-mCherry localization in the absence of SpoVQ (Fig. 6), SpoVQ would appear to require access to SipL’s C-terminal LysM domain. Thus, if SipL’s LysM domain binds peptidoglycan, SipL could provide a physical link between coat and cortex formation similar to how SafA “staples” the outer forespore membrane to the cortex (45). Addressing the accessibility of SpoVQ and SipL’s C-terminal domains to protease treatment in sporulating cells that have not completed engulfment would provide critical insight into these questions. However, these types of analyses will likely be challenging due to C. difficile’s S-layer reducing access to proteases and lysozyme (56), as well as the intrinsic resistance of its cell wall to lysozyme digestion (57).

Finally, another key question raised by our study is how SpoVQ specifically localizes to the outer forespore membrane. *spoVQ* expression is controlled by the mother cell-specific sigma factor, σ^E^ (30, 31), so SpoVQ could localize to all mother cell-derived membranes. However, we found that SpoVQ-mCherry localized to the polar septum during asymmetric division (Fig. 5), so preferential localization to forespore membranes appears to occur early during spore formation (Fig. 5). This localization is independent of whether the basement layer coat proteins, SpoIVA or SipL, are present, although slightly more cytosolic signal is observed in the absence of SpoIVA (Fig. 5). These results contrast with the localization requirements for B. subtilis SsdC, whose preferential localization to the outer forespore membrane depends on basement layer coat proteins (50). SsdC also exhibits a different localization pattern than SpoVQ, since SsdC forms two distinct foci at the mother cell-forespore interface. Analyzing the localization dependence of SpoVQ-mCherry in mutants defective in engulfment, SpoIIQ-SpoIIIAH channel components, or cortex synthesis would provide insight into how SpoVQ specifically concentrates in the outer forespore membrane.

Taken together, considerable work remains to understand how the *Peptostreptococcaceae* family-specific spore morphogenetic protein SpoVQ regulates C. difficile cortex synthesis in concert with the coat morphogenetic proteins, SpoIVA and SipL. Regardless, our identification of this unique regulator reveals for the first time a direct link between coat localization and cortex synthesis in C. difficile. This work, along with recent work in B. subtilis (45, 50) suggests that the direct coordination of these two processes may be universally conserved across spore-forming bacteria even if the specific regulators and their mechanism of action may differ.

## MATERIALS AND METHODS

### Bacterial strains and growth conditions.

The C. difficile strains used are listed in Table S1 (supplemental materials). All strains derive from the erythromycin-sensitive 630*ΔermΔpyrE* parental strain, which is a sequenced clinical isolate 630, which we used for *pyrE-*based allele-coupled exchange (ACE) (29). Strains were grown on BHIS (brain heart infusion supplemented with yeast extract and cysteine [58]) and taurocholate (TA; 0.1%, [wt/vol]; 1.9 mM), cefoxitin (8 μg/ml), and kanamycin (50 μg/ml) as needed. For ACE, C. difficile defined medium (59) (CDDM) was supplemented with 5-fluorootic acid (5-FOA; at 2 mg/ml) and uracil (at 5 μg/ml).

10.1128/mSphere.00211-21.1 TABLE S1 Strains used in this study. Download Table S1, DOCX file, 0.03 MB. Copyright © 2021 Touchette et al. 2021 Touchette et al. https://creativecommons.org/licenses/by/4.0/ This content is distributed under the terms of the Creative Commons Attribution 4.0 International license.

The Escherichia coli strains used for HB101/pRK24-based conjugations and BL21(DE3) expression analyses are listed in Table S1. E. coli strains were grown at 37°C with shaking at 225 rpm in Luria-Bertani (LB) broth. The medium was supplemented with ampicillin (50 μg/ml), chloramphenicol (20 μg/ml), or kanamycin (30 μg/ml) as needed.

### E. coli strain construction.

All primers used for cloning are listed in Table S2. Details of E. coli strain construction are provided in Text S1. Plasmid constructs were confirmed by sequencing using Genewiz and transformed into either the HB101/pRK24 conjugation strain (used with C. difficile) or the BL21(DE3) expression strain.

10.1128/mSphere.00211-21.2 TABLE S2 Primers used in this study. Download Table S2, DOCX file, 0.02 MB. Copyright © 2021 Touchette et al. 2021 Touchette et al. https://creativecommons.org/licenses/by/4.0/ This content is distributed under the terms of the Creative Commons Attribution 4.0 International license.

10.1128/mSphere.00211-21.3 TEXT S1 Detailed cloning description of E. coli strain construction. Download Text S1, DOCX file, 0.03 MB. Copyright © 2021 Touchette et al. 2021 Touchette et al. https://creativecommons.org/licenses/by/4.0/ This content is distributed under the terms of the Creative Commons Attribution 4.0 International license.

### C. difficile strain construction and complementation.

Allele-couple exchange (ACE) (29) was used to construct all complementation strains. ACE was also used to introduce the *sipL* deletion using strain no. 1704 pMTL-YN3 Δ*sipL* into 630Δ*erm*Δ*pyrE spoIVA* ATPase mutants. Complementations were performed as previously described by conjugating HB101/pRK24 carrying pMTL-YN1C plasmids into Δ*pyrE-*based strains (60) using allele-coupled exchange.

### Plate-based sporulation assay.

C. difficile strains were grown overnight from glycerol stocks on BHIS plates containing 0.1% (wt/vol) taurocholate. Colonies from these plates were inoculated into BHIS liquid medium and then back-diluted 1:25 once they were in stationary phase. The cultures were grown until they reached an optical density (OD_600nm_) between 0.4 and 0.7, and 120 μl was used to inoculate 70:30 agar plates (20). Sporulation was induced for 20 to 24 h, after which cells were analyzed by phase-contrast microscopy to assess sporulation levels and harvested for Western blot analyses, and the proportion of heat-resistant spores was measured.

### Immunoprecipitation analyses.

Immunoprecipitations were performed on lysates prepared from cultures induced to sporulate on 70:30 plates for 24 h. The samples were processed as previously described (25), although for some of the replicates, anti-FLAG magnetic beads (Sigma-Aldrich) were used to pull down FLAG-tagged proteins and any associated proteins rather than the Dynabead protein G (Invitrogen) prebound with anti-FLAG M2 antibody (Sigma-Aldrich) as described in reference 25. Where indicated, NP40 detergent was added at 0.1% (vol/vol) to the FLAG-immunoprecipitation (IP) buffer (FIB) (50 mM Tris, pH 7.5, 150 mM NaCl, 0.02% NaN_3_, 1× protease inhibitor (HALT cocktail; Thermo Scientific) after the lysate was prepared following cell lysis via bead-beating (FastPrep Pro; MP Biomedicals). Immunoprecipitations were performed on 2 to 3 biological replicates.

For immunoprecipitation experiments coupled to quantitative mass spectrometry-based proteomics, duplicates of each *ΔsipL*/*sipL* and Δ*sipL*/*sipL-FLAG*_3_ strain were prepared simultaneously. They were induced to sporulate for 24 h, and the samples were prepared as previously described (25) using magnetic Dynabead protein G (Invitrogen) coupled to anti-FLAG M2 antibody (Sigma-Aldrich). Immunoprecipitations were carried out for 2 h at room temperature and the beads were washed four times with FIB. Efficient immunoprecipitation was confirmed by Western blotting prior to proceeding with mass spectrometry.

The beads from the immunoprecipitations were washed an additional four times with phosphate-buffered saline (PBS). The four different lysates of each replicate were resuspended in 90 μl digestion buffer (2 M urea, 50 mM Tris HCl), and 2 μg of sequencing-grade trypsin was added and shaken for 1 h at 700 rpm. The supernatant was removed and placed in a fresh tube. The beads were then washed twice with 50 μl digestion buffer and combined with the supernatant. The combined supernatants were reduced (2 μl of 500 mM dithiothreitol [DTT], 30 min, room temperature [RT]), alkylated (4 μl of 500 mM iodoacetamide (IAA), 45 min, dark), and a longer overnight digestion was performed as follows: 2 μg (4 μl) trypsin and shaken overnight. The samples were then quenched with 20 μl of 10% formic acid and desalted on 10 mg Oasis cartridges. Desalted peptides from each pulldown were separately labeled with a specific iTRAQ4 mass tagging reagent (lot A7024; Sciex) prior to mixing and analysis by LC-MS/MS. The tag layout of the iTRAQ 4-plex was as follows: 114, control (ΔsipL/sipL) rep 1; 115 control rep 2; 116, experimental (ΔsipL/sipL-FLAG3) rep 1; 117, SIPL rep 2. Peptides were dissolved in 50 μl of 100% ethanol, and one kit of iTRAQ reagent was added to each vial according to the lot information. The reaction proceeded for 1 h at room temperature. The resulting pool of labeled peptides was dried and separated into six fractions using high-pH (pH 10) fractionation on an SDB STAGE (stop and go extraction) tip with 4 punches (Empore reversed phase extraction disks from 3M, SDB-XC reversed phase material, product number 2240/2340). The six fractions were as follows: fraction 0, 3% acetonitrile; fraction 1, 5% acetonitrile; fraction 2, 10% acetonitrile; fraction 3, 15% acetonitrile; fraction 4, 20% acetonitrile; fraction 5, 30% acetonitrile; fraction 6, 50% acetonitrile.

Reconstituted peptides were separated on an online nanoflow EASY-nLC 1000 ultra-high-performance liquid chromatography (UHPLC) system (Thermo Fisher Scientific) and analyzed on a benchtop Orbitrap Q Exactive Plus mass spectrometer (Thermo Fisher Scientific). The peptide samples were injected onto a capillary column (Picofrit with 10 μm tip opening/75 μm diameter; New Objective, PF360–75-10-N-5) packed in-house with 20 cm C_18_ silica material (1.9 μm ReproSil-Pur C18-AQ medium; Dr. Maisch GmbH), and heated to 50°C in column heater sleeves (Phoenix-ST) to reduce backpressure during UHPLC separation. Injected peptides were separated at a flow rate of 200 nl/min with a linear 230-min gradient from 100% solvent A (3% acetonitrile, 0.1% formic acid) to 30% solvent B (90% acetonitrile, 0.1% formic acid), followed by a linear 9-min gradient from 30% solvent B to 60% solvent B and a 1-min ramp to 90% B. Each sample fraction was run for 120 min, including sample loading and column equilibration times. The Q Exactive instrument was operated in the data-dependent mode acquiring high cell density (HCD) MS/MS scans (*R* = 17,500) after each MS1 scan (*R* = 70,000) on the 12 top most abundant ions using an MS1 ion target of 3 × 10^6^ ions and an MS2 target of 5 × 10^4^ ions. The maximum ion time utilized for the MS/MS scans was 120 ms; the HCD-normalized collision energy was set to 27; the dynamic exclusion time was set to 20 s, and the peptide match and isotope exclusion functions were enabled.

All mass spectra were processed using the Spectrum Mill software package v6.0 prerelease (Agilent Technologies), which includes modules developed by us for iTRAQ4-based quantification. For peptide identification, MS/MS spectra were searched against the human UniProt database, to which a set of common laboratory contaminant proteins was appended. Search parameters included ESI-Q EXACTIVE-HCD scoring parameters, trypsin enzyme specificity with a maximum of two missed cleavages, 40% minimum matched peak intensity, ±20 ppm precursor mass tolerance, ±20 ppm product mass tolerance, and carbamidomethylation of cysteines and iTRAQ4 labeling of lysines and peptide n-termini as fixed modifications. Variable modifications that were allowed included oxidation of methionine, N-terminal acetylation, pyroglutamic acid (N-termQ), deamidated (N), pyro carbamidomethyl Cys (N-termC), with a precursor MH+ shift range of –18 to 64 Da. Identities interpreted for individual spectra were automatically designated valid by optimizing score and delta rank1-rank2 score thresholds separately for each precursor charge state in each LC-MS/MS while allowing a maximum target-decoy-based false-discovery rate (FDR) of 1.0% at the spectrum level.

The expectation maximization algorithm was applied to the results of the peptide report. The list of most likely observed proteins was generated for each channel of the MS experiment based on the Swiss-Prot and TrEMBLE databases of protein sequences. Next, ratios of intensities between channels were calculated and median normalized. The resulting data were analyzed and visualized using R. Statistical analyses were performed via moderated *t* test from the R package limma to estimate *P* values for each protein, and the false-discovery rate corrections (FDR) were applied to account for multiple hypothesis testing. Plots were created using in-house-written R scripts and gplot2. Hits were defined as proteins with a ≥2-fold enrichment with a *P* value of <0.01.

### Heat resistance assay on sporulating cells.

Heat-resistant spore formation was assessed 20 to 24 h after sporulation induction as previously described (33). Heat-resistance efficiencies represent the average ratio of heat-resistant CFU obtained from spores in a given sample relative to the average ratio determined for the wild type based on a minimum of three biological replicates. Statistical significance was determined using one-way ANOVA and Tukey’s test.

### Western-blot analyses.

Samples for Western-blotting analyses were prepared as previously described (20). Briefly, sporulating cell pellets were resuspended in 100 μl of PBS and subjected to three freeze-thaw cycles. Denaturing EBB buffer was added to the sample at an ∼1:1 ratio (8 M urea, 2 M thiourea, 4% [wt/vol] SDS, 2% [vol/vol] β-mercaptoethanol). The samples were boiled for 20 min, vortexed, pelleted at high-speed, and resuspended in the same supernatant to maximize protein solubilization. Finally, the samples were boiled for 5 min and pelleted again at high-speed. Samples were resolved on SDS-PAGE gels, transferred to Immobilon-FL polyvinylidene difluoride (PVDF) membranes, and blocked in Odyssey blocking buffer with 0.1% (vol/vol) Tween 20. Rabbit anti-SpoVQ_Δ32_ (this study) and anti-CPD (46) antibodies were used at a 1:1,000 dilution. Rabbit anti-SipL_ΔLysM_ and mouse anti-SpoIVA (61) antibodies were used at a 1:2,500 dilution. Rabbit anti-mCherry (Abcam, Inc.) was used at a 1:2,000 dilution. IRDye 680CW and 800CW infrared dye-conjugated secondary antibodies were used at a 1:20,000 dilution, and blots were imaged on an Odyssey LiCor CLx imaging system.

### Spore purification.

Sporulation was induced in 70:30 agar plates for 2 to 3 days as previously described (18, 62). C. difficile sporulating cultures were lysed in 5 to 6 cycles of ice-cold water washes, incubated on ice overnight at 4°C, pelleted, and treated with DNase I (New England Biolabs) at 37°C for 60 min. Finally, spores were purified on a 20%:50% HistoDenz gradient (Sigma-Aldrich) and resuspended in water. Spore purity was determined by phase-contrast microscopy (>95%), and the optical density of the spore preparation was measured at OD_600_. Spore yields were quantified by measuring the OD_600nm_ of the spore purifications from four 70:30 plates per replicate. The average of six biological replicates was calculated, and statistical significance was determined using a one-way analysis of variance (ANOVA) and Tukey’s test. Spores were stored in water at 4°C.

### Germination assays.

Germination assays were performed as previously described (63). Approximately, ∼1 × 10^7^ spores (0.35 OD_600_ units) were resuspended in 100 μl of water, and 10 μl of this mixture was removed for 10-fold serial dilutions in PBS. The dilutions were plated on either BHIS alone or BHIS containing 0.1% taurocholate germinant. Colonies arising from germinated spores were enumerated after 18 to 24 h. Germination efficiencies were calculated by averaging the CFU produced by spores for a given strain relative to the number produced by wild-type spores from at least three independent spore preparations. Statistical significance was determined by performing a one-way analysis of variance (ANOVA) on natural log-transformed data using Tukey’s test. The data were transformed because the use of independent spore preparations resulted in a nonnormal distribution for spontaneous germination results.

### TEM analysis.

Sporulating cultures (23 to 24 h) were fixed and processed for electron microscopy by the University of Vermont Microscopy Center according to previously published protocols (20). A minimum of 50 full-length sporulating cells were used for phenotype counting.

### mCherry fluorescence microscopy.

Live-cell fluorescence microscopy was performed using Hoechst 33342 (15 μg/ml; Molecular Probes) and mCherry protein fusions. Samples were prepared on agarose pads using Gene Frames (Thermo Scientific) as previously described (23). Images were taken 30 min after harvesting the C. difficile sporulating cultures to allow the mCherry fluorescent signal to reconstitute under aerobic conditions.

Phase-contrast and fluorescence microscopy were carried out on a Nikon ×60 oil immersion objective (1.4 numerical aperture [NA]) using a Nikon 90i epifluorescence microscope. A CoolSnap HQ camera (Photometrics) was used to acquire multiple fields for each sample in 12-bit format with 2-by-2 binning, using NIS-Elements software (Nikon). The Texas red channel was used to acquire images after 300 ms for mCherry-SpoIVA, 100 ms for SipL-mCherry, and 400 ms for CotE-mCherry. Hoechst stain was visualized using 90-ms exposure time, and 3-ms exposure was used for phase-contrast pictures. Finally, 10 MHz images were imported to Adobe Photoshop CC 2019 software for pseudocoloring and minimal adjustments in brightness and contrast levels. Protein localization analyses were performed on a minimum of three independent biological replicates.

### Protein purification for antibody production and coaffinity purification analyses.

His_6_-tagged proteins were affinity-purified from E. coli BL21(DE3) cultures (see Table S2) grown in 2YT medium (5 g NaCl, 10 g yeast extract, and 15 g tryptone per liter) after an overnight induction with 250 μM IPTG (isopropyl-β-d-thiogalactopyranoside) at 19°C as previously described (62). The anti-SpoVQ antibody was raised against SpoVQ_Δ32_-His_6_, which lacks its transmembrane domain (first 32 codons), and the antibody against CotL was raised against CotL-His_6_ in rabbits by Cocalico Biologicals (Reamstown, PA).

Cultures were pelleted, resuspended in lysis buffer (500 mM NaCl, 50 mM Tris [pH 7.5], 15 mM imidazole, 10% [vol/vol] glycerol), flash-frozen in liquid nitrogen, and sonicated. The insoluble material was pelleted, and the soluble fraction was incubated with Ni-NTA agarose beads (0.5 ml; Qiagen) for 3 h and eluted using high-imidazole buffer (500 mM NaCl, 50 mM Tris [pH 7.5], 200 mM imidazole, 10% [vol/vol] glycerol) after nutating the sample for 5 to 10 min. The resulting induction and eluate fractions were separated by SDS-PAGE and analyzed by Western blotting as described above.

For the coaffinity purification assays, E. coli BL21(DE3) strains were simultaneously transformed with two plasmids to express combinations of SpoVQ_Δ32_-His_6_ and either untagged SpoIVA, SipL, or CPD or empty vector. Coaffinity purifications were performed using Ni^2+^-affinity resin as described above.
