# Supplementary material for: Identification of a Novel Regulator of Clostridioides difficile Cortex Formation
Source: mSphere. 2021 May 28;6(3):e00211-21. doi: 10.1128/mSphere.00211-21 (PMC8265636; doi:10.1128/mSphere.00211-21)
Supplement: TABLE S1 [file msphere.00211-21-st001.docx]

**Supplementary Table S1. Strains used in this study**

| **Strain**  **#** | **Strain name** | **Relevant genotype or features** | | **Source/reference** | | | |
| --- | --- | --- | --- | --- | --- | --- | --- |
| ***C. difficile* strains – 630∆*erm*** | | | | |  | |  |
| 803 | 630∆*erm*∆*pyrE* ∆*spoIVA* | | 630∆*erm* ∆*pyrE* with *spoIVA* (*CD2629*) deleted | | | (1) |  |
| 846 | 630∆*erm*-p | | *erm*-sensitive derivate of 630 with *pyrE* restored | | | (2) |  |
| 849 | 630∆*erm ∆spo0A*-p | | 630∆*erm* ∆*spo0A* with *pyrE* restored | | | (2) |  |
| 880 | 630∆*erm ∆spoIVA*-p | | 630∆*erm* ∆*spoIVA* with *pyrE* restored | | | (1) |  |
| 925 | 630∆*erm ∆sleC*-p | | 630∆*erm* ∆*sleC* with *pyrE* restored | | | (2) |  |
| 1005 | 630∆*erm*∆*pyrE* ∆*sipL* | | 630∆*erm*∆*pyrE* with *sipL* deleted | | | (3) |  |
| 1010 | 630∆*erm* ∆*sipL*-p | | 630∆*erm* with *sipL* deleted and *pyrE* restored | | | (3) |  |
| 1013 | 630∆*erm* ∆*sipL*/*sipL* | | 630∆*erm* ∆*sipL* with *sipL* in the *pyrE* locus | | | (3) |  |
| 1144 | 630∆*erm*/*mCherry*-*IVA* | | 630∆*erm* with *mCherry-IVA* in the *pyrE* locus | | | (1) |  |
| 1158 | 630∆*erm ∆sipL*/*sipL-mCherry* | | 630∆*erm* ∆*sipL* with *sipL-mCherry* in the *pyrE* locus | | | (3) |  |
| 1238 | 630∆*erm ∆cspC-p* | | 630∆*erm* ∆*cspC* with *pyrE* restored | | | (2) |  |
| 1377 | 630∆*erm ∆sipL*/*sipL-FLAG_3_* | | 630∆*erm ∆sipL* with *sipL-FLAG_3_* in the *pyrE* locus | | | (4) |  |
| 1797 | 630∆*erm ∆spoVQ*∆*pyrE* | | 630∆*erm* ∆*pyrE* with *spoVQ* deleted | | | This study |  |
| 1804 | 630∆*erm ∆spoVQ-p* | | 630∆*erm* ∆*spoVQ* with *pyrE* restored | | | This study |  |
| 1807 | 630∆*erm ∆spoVQ/spoVQ* | | 630∆*erm* ∆*spoVQ* with *spoVQ* in the *pyrE* locus | | | This study |  |
| 1810 | 630∆*erm ∆spoVQ/spoVQ_-_FLAG_3_* | | 630∆*erm* ∆*spoVQ* with *spoVQ-FLAG_3_* in the *pyrE* locus | | | This study |  |
| 1813 | 630∆*erm* ∆*spoVQ/spoVQ-mCherry* | | 630∆*erm* ∆*spoVQ* with *spoVQ-mCherry* in the *pyrE* locus | | | This study |  |
| 1825 | 630∆*erm ∆spoVQ/spoVQ_∆32_* | | 630∆*erm* ∆*spoVQ* with *spoVQ_∆32_* in the *pyrE* locus | | | This study |  |
| 1888 | 630∆*erm ∆spoVQ*/*mCherry-IVA* | | 630∆*erm ∆spoVQ* with *mCherry-spoIVA* in the *pyrE* locus | | | This study |  |
| 1929 | 630∆*erm* ∆*cspC∆spoVQ*∆*pyrE* | | 630∆*erm* ∆*cspC∆pyrE* with *spoVQ* deleted | | | This study |  |
| 1932 | 630∆*erm* ∆*sleC∆spoVQ*∆*pyrE* | | 630∆*erm* ∆*sleC∆pyrE* with *spoVQ* deleted | | | This study |  |
| 1935 | 630∆*erm* ∆*sipL∆spoVQ*∆*pyrE* | | 630∆*erm* ∆*sipL∆pyrE* with *spoVQ* deleted | | | This study |  |
| 1962 | 630∆*erm ∆sleC*∆*spoVQ*-p | | 630∆*erm* ∆*sleC∆spoVQ* with *pyrE* restored | | | This study |  |
| 1965 | 630∆*erm ∆sipL*∆*spoVQ*-p | | 630∆*erm* ∆*sipL∆spoVQ* with *pyrE* restored | | | This study |  |
| 1968 | 630∆*erm ∆sipL*∆*spoVQ*/*sipL-mCherry* | | 630∆*erm* ∆*sipL∆spoVQ* with *sipL-mCherry* in the *pyrE* locus | | | This study |  |
| 1977 | 630∆*erm ∆cspC*∆*spoVQ*-p | | 630∆*erm* ∆*cspC∆spoVQ* with *pyrE* restored | | | This study |  |
| 1995 | 630∆*erm ∆cspC*∆*spoVQ*/*spoVQ* | | 630∆*erm* ∆*cspC∆spoVQ* with *spoVQ* in the *pyrE* locus | | | This study |  |
| 1998 | 630∆*erm ∆sleC*∆*spoVQ*/*spoVQ* | | 630∆*erm* ∆*sleC∆spoVQ* with *spoVQ* in the *pyrE* locus | | | This study |  |
| 2017 | 630∆*erm* ∆*spoIVA∆spoVQ*∆*pyrE* | | 630∆*erm* ∆*spoIVA∆pyrE* with *spoVQ* deleted | | |  |  |
| 2113 | 630∆*erm ∆spoIVA*∆*spoVQ*-p | | 630∆*erm* ∆*spoIVA∆spoVQ* with *pyrE* restored | | | This study |  |
| 2242 | 630∆*erm*∆*pyrE* ∆*cotL* | | 630∆*erm*∆*pyrE* with *cotL* deleted | | | This study |  |
| 2271 | 630∆*erm ∆cotL/cotL* | | 630∆*erm* ∆*cotL* with with *cotL* in the *pyrE* locus | | | This study |  |
| 2277 | 630∆*erm ∆cotL-p* | | 630∆*erm* ∆*cotL* with *pyrE* restored | | | This study |  |
| 2318 | 630∆*erm*/*spoVQ-mCherry* | | 630∆*erm* with *spoVQ-mCherry* in the *pyrE* locus | | | This study |  |
| 2321 | 630∆*erm ∆sipL*∆*spoVQ*/*spoVQ-mCherry* | | 630∆*erm* ∆*sipL∆spoVQ* with *spoVQ-mCherry* in the *pyrE* locus | | | This study |  |
| 2324 | 630∆*erm ∆spoIVA*∆*spoVQ*/*spoVQ-mCherry* | | 630∆*erm* ∆*spoIVA∆spoVQ* with *spoVQ-mCherry* in the *pyrE* locus | | | This study |  |
| 2442 | 630∆*erm ∆sipL*/*mCherry-sipL* | | 630∆*erm* ∆*sipL* with *mCherry-sipL* in the *pyrE* locus | | | This study |  |
| 2827 | 630∆*erm ∆sipL*∆*spoVQ*/*mCherry-sipL* | | 630∆*erm* ∆*sipL∆spoVQ* with *mCherry-sipL* in the *pyrE* locus | | | This study |  |
|  |  | |  | | |  |  |
| ***E. coli* strains** | | |  | | |  |  |
| **Strain #** | **Strain name** | | **Relevant genotype or features** | | | **Source** |  |
| 41 | DH5α | | F– Φ80*lacZ*ΔM15 Δ(*lacZYA-argF*) U169 *recA1 endA1 hsdR17* (rK^–^, mK^+^) *phoA supE44* λ– *thi-1 gyrA96 relA1* | | | D. Cameron |  |
| 531 | HB101/pRK24 | | F- *mcrB mrr hsdS20*(rB^–^mB^–^) *recA13 leuB6 ara-13 proA2 lavYI galK2 xyl-6 mtl-1 rpsL20* carrying pRK24 | | | C. Ellermeier |  |
| 903 | BL21(DE3) pET28a + pET21a *sipL*(TAA) | | pET28a + pET21a *sipL*(TAA) | | |  |  |
| 1768 | HB101 pMTL-YN1C *mCherry-spoIVA* | | pMTL-YN1C *mCherry-spoIVA* | | | (1) |  |
| 1777 | HB101 pMTL-YN1C *sipL-mCherry* | | pMTL-YN1C *sipL-mCherry* in HB101 | | | (3) |  |
| 642 | DH5α pET21a-*spoIVA*(TAA) | | pET21a-*spoIVA*(TAA) | | | (5) |  |
| 643 | DH5α pET21a-*sipL*(TAA) | | pET21a-*sipL*(TAA) | | | (5) |  |
| 1975 | DH5α pET28a-*spoVQ*-His_6_ | | pET28a-*spoVQ*_∆32_-His_6_ | | | This study |  |
| 1976 | BL21(DE3) pET28a-*spoVQ*-His_6_ | | pET28a-*spoVQ*-His_6_ | | | This study |  |
| 1978 | BL21(DE3) pET28a-*spoVQ*_∆32_-His_6_ | | pET28a-*spoVQ*_∆32_-His_6_ | | | This study |  |
| 1996 | HB101 pMTL-YN3 ∆*spoVQ* | | pMTL-YN3 ∆*spoVQ* in HB101 | | | This study |  |
| 2050 | HB101 pMTL-YN1C *spoVQ* | | pMTL-YN1C *spoVQ* in HB101 | | | This study |  |
| 2052 | HB101 pMTL-YN1C *spoVQ-FLAG_3_* | | pMTL-YN1C *spoVQ-FLAG_3_* in HB101 | | | This study |  |
| 2054 | HB101 pMTL-YN1C *spoVQ-mCherry* | | pMTL-YN1C *spoVQ-mCherry* in HB101 | | | This study |  |
| 2056 | HB101 pMTL-YN1C *spoVQ*_∆32_ | | pMTL-YN1C *spoVQ*_∆32_ in HB101 | | | This study |  |
| 2355 | HB101 pMTL-YN1C *mCherry-sipL* | | pMTL-YN1C *mCherry-sipL* in HB101 | | | This study |  |
| 2067 | BL21(DE3) pET22b-CPD(TAA) | | pET22b-CPD(TAA) | | | This study |  |
| 2100 | BL21(DE3) pET28a-*spoVQ*_∆32_-His_6_ + pET21a | | pET28a-*spoVQ*_∆32_-His_6_ + pET21a | | | This study |  |
| 2057 | BL21(DE3) pET28a-*spoVQ*_∆32_-His_6_ + pET21a-*sipL*(TAA) | | pET28a-*spoVQ*_∆32_-His_6_ + pET21a-*sipL*(TAA) | | | This study |  |
| 2058 | BL21(DE3) pET28a-*spoVQ*_∆32_-His_6_ + pET21a-*spoIVA*(TAA) | | pET28a-*spoVQ*_∆32_-His_6_ + pET21a-*spoIVA*(TAA) | | | This study |  |
| 2071 | BL21(DE3) pET28a-*spoVQ*_∆32_-His_6_ + pET21a-CPD(TAA) | | pET28a-*spoVQ*_∆32_-His_6_ + pET21a-CPD(TAA) | | | This study |  |
| 2102 | BL21(DE3) pET28a + pET21a-CPD(TAA) | | pET28a + pET21a-CPD(TAA) | | | This study |  |
| 2239 | pMTL-YN3-∆*cotL* | | pMTL-YN3 ∆*cotL* in HB101 | | | This study |  |
| 2278 | pET22b-*cotL* | | pET22b-*cotL* in BL21(DE3) | | | This study |  |
| 2261 | pMTL-YN1C-*cotL* | | pMTL-YN1C *cotL* in HB101 | | | This study |  |
| 2354 | pMTL-YN1C *mCherry-sipL* | | pMTL-YN1C *mCherry-sipL in* | | | This study |  |

**Plasmids**

| pET28a | For cloning His-tagged expression constructs | Novagen |
| --- | --- | --- |
| pET21a | For cloning His-tagged expression constructs | Novagen |
| pMTL-YN1C | For cloning complementation constructs to be integrated into the pyrE locus of 630∆*erm*∆*pyrE* | (6) |
| pMTL-YN3 | For cloning allelic exchange constructs to modify 630∆*erm*∆*pyrE* | (6) |

**References**

1. Ribis JW, Ravichandran P, Putnam EE, Pishdadian K, Shen A. 2017. The Conserved Spore Coat Protein SpoVM Is Largely Dispensable in *Clostridium difficile* Spore Formation. mSphere 2.

2. Donnelly ML, Li W, Li YQ, Hinkel L, Setlow P, Shen A. 2017. A *Clostridium difficile*-Specific, Gel-Forming Protein Required for Optimal Spore Germination. mBio 8.

3. Ribis JW, Fimlaid KA, Shen A. 2018. Differential requirements for conserved peptidoglycan remodeling enzymes during *Clostridioides difficile* spore formation. Mol Microbiol 110:370-389.

4. Touchette MH, Benito de la Puebla H, Ravichandran P, Shen A. 2019. SpoIVA-SipL Complex Formation Is Essential for *Clostridioides difficile* Spore Assembly. J Bacteriol 201.

5. Putnam EE, Nock AM, Lawley TD, Shen A. 2013. SpoIVA and SipL are *Clostridium difficile* spore morphogenetic proteins. J Bacteriol 195:1214-25.

6. Ng YK, Ehsaan M, Philip S, Collery MM, Janoir C, Collignon A, Cartman ST, Minton NP. 2013. Expanding the repertoire of gene tools for precise manipulation of the *Clostridium difficile* genome: allelic exchange using *pyrE* alleles. PLoS One 8:e56051.
