# Supplementary material for: Identification of a Novel Regulator of Clostridioides difficile Cortex Formation
Source: mSphere. 2021 May 28;6(3):e00211-21. doi: 10.1128/mSphere.00211-21 (PMC8265636; doi:10.1128/mSphere.00211-21)
Supplement: TABLE S2 [file msphere.00211-21-st002.docx]

**Table S2. Primers used in this study.**

| **Number** | **Primer Name** | **Primer Sequence** |
| --- | --- | --- |
| 2133 | 3' XhoI *mCherry* Gibson | gccaagcttgcatgtctgcaggcCTCGAGTTATTTATATAATTCATCCATACCTCCTGTTG |
| 2462 | 5' NcoI *spoVQ* | AGCCCATGGCATTGAATGTAAAGTTTAATATTAAAGGTATAATTTATG |
| 2463 | 3' XhoI *spoVQ* | GAGCCTCGAGTTTCGTTGCATTTAATTCTATTCTATCTGGAAG |
| 2464 | 5' NcoI *spoVQ*_∆32_ | AGCCCATGGCATTTGTAGAGAAATCTAAACCCATAGATTATACAG |
| 2484 | 5' AscI ∆*spoVQ* Gibson | gtcaattgttcaaaaaaataatggcGGCGCGCCTTATGGCATTCTTAACTGGATTAGGAG |
| 2485 | 5' ∆*spoVQ* SOE | GAGTTATAGCTCTTGTATTCGTTATAGGGGAACTTCCAGATAGAATAGAATTAAATGCA |
| 2486 | 3' ∆*spoVQ* rev eos | TGCATTTAATTCTATTCTATCTGGAAGTTCCCCTATAACGAATACAAGAGCTATAACTC |
| 2487 | 3' SbfI ∆*spoVQ* Gibson | gcaaggcaagaccgatcgggcccCCTGCAGGCTTCAAATGGAAGATATAAGAAATGCAAC |
| 2540 | 5' NotI *spoVQ* Gibson | ggaattagggatgtaataagcggccgcCAAATAAGTATTTTTTAATATTGTAAAC |
| 2541 | 3' *spoVQ* mCherry eos | CTTCTTCTCCTTTAGATACCATTGCTTTCGTTGCATTTAATTCTATTC |
| 2542 | 5' mCherry *spoVQ* SOE | GAATAGAATTAAATGCAACGAAAGCAATGGTATCTAAAGGAGAAGAAG |
| 2543 | 3' XhoI *spoVQ* Gibson | ttgcatgtctgcaggcctcgagTTATTTCGTTGCATTTAATTCTATTC |
| 2544 | 3' mid *spoVQ* for FLAG | CTTTCTATTTTGTCCATTTCTATACCATATTTATTATTTCCTCTAGTCAC |
| 2549 | 3' 5'UTR *spoVQ_∆32_* rev | CTATGGGTTTAGATTTCTCTACAAATGCCATTCTTATCACTCCTCCCATATATTC |
| 2550 | 5' YN1C *spoVQ_∆32_* SOE | GAATATATGGGAGGAGTGATAAGAATGGCATTTGTAGAGAAATCTAAACCCATAG |
| 2573 | 5' NdeI CPD 22b | TTTGTTTAACTTTAAGAAGGAGATATACATATGGCATTAGCGGATGGAAAAATACTCC |
| 2574 | 3' XhoI CPD+TAA 22b | CAGTGGTGGTGGTGGTGGTGCTCGAGTTAACCTTGCGCGTCCCAGCTTAGCGAAAC |
| 2820 | 5' AscI ∆*cotL* Gibson | gtcaattgttcaaaaaaataatggcggcgcgccGAGATTATTCATACTACCAAGATTG |
| 2821 | 5' ∆*cotL* SOE | TGAATATAATTCAATATATATTTATAGAAAGGGGTAAAACTAAAAAGTCTAACCCTCATG |
| 2822 | 3' ∆*cotL* rev eos | CATGAGGGTTAGACTTTTTAGTTTTACCCCTTTCTATAAATATATATTGAATTATATTCA |
| 2823 | 3' SbfI ∆*cotL* Gibson | agcaaggcaagaccgatcgggccccctgcaggGAGTATAAATCTATTGAGAGC |
| 2833 | 5' NotI *cotL* Gibson | aattagggatgtaataagcggccgcCTTAAATTGCCATGTGTAGACTGTG |
| 2834 | 3' XhoI *cotL* Gibson | caagcttgcatgtctgcaggcctcgagCTATTCATGAGGGTTAGACTTTTTAG |
| 2946 | 5’ NdeI *cotL* pET22b | TTTAACTTTAAGAAGGAGATATACATATGCTTAAATTGCCATGTGTAGACTGTGAAAG |
| 2947 | 3’ XhoI *cotL* pET22b | ATCTCAGTGGTGGTGGTGGTGGTGCTCGAGTTCATGAGGGTTAGACTTTTTAGTTTTC |
| 3019 | 5’ *mCherry-sipL* SOE | CAGGAGGTATGGATGAATTATATAAAGCAATGGAATTAATTAAAGATGTAATTAAAG |
| 3020 | 3’ *mCherry-sipL* EOS | CTTTAATTACATCTTTAATTAATTCCATTGCTTTATATAATTCATCCATACCTCCTG |
| 3021 | 5’ P*sipL*-*mCherry* SOE | ATATTTTTATAATACTTAAGGAGGTAGACTATGGTATCTAAAGGAGAAGAAGATAATATG |
| 3022 | 3’ P*sipL*-*mCherry* EOS | CATATTATCTTCTTCTCCTTTAGATACCATAGTCTACCTCCTTAAGTATTATAAAAATAT |

Restriction sites are underlined.
