# Supplementary material for: Identification of a Novel Regulator of Clostridioides difficile Cortex Formation
Source: mSphere. 2021 May 28;6(3):e00211-21. doi: 10.1128/mSphere.00211-21 (PMC8265636; doi:10.1128/mSphere.00211-21)
Supplement: TEXT S1 [file msphere.00211-21-t0001.docx]

**Supplementary Text**

***E. coli* strain construction**

**pET28a-*spoVQ*-His_6_**. To clone full-length His-tagged SpoVQ for protein expression in *E. coli*, primer pair #2642 and 2643 were used to amplify the *spoVQ* gene lacking its stop codon. The resulting PCR product was digested with NcoI and XhoI and ligated into pET28a digested with the same enzymes. The ligation was transformed into DH5α, and the resulting construct was sequenced verified before transforming the plasmid into BL21(DE3) for protein expression.

**pET28a-*spoVQ_∆_*_32_-His_6_**. To clone His-tagged SpoVQ lacking its N-terminal transmembrane domain (codons 1-32) for protein expression in *E. coli*, primer pair #2464 and 2465were used to amplify the *spoVQ* gene lacking its stop codon and first 32 codons. The resulting PCR product was digested with NcoI and XhoI and ligated into pET28a digested with the same enzymes. The ligation was transformed into DH5α, and the resulting construct was sequenced verified before transforming the plasmid into BL21(DE3) for protein expression.

**pET22b-*cotL*-His_6_**. To clone full-length His-tagged CotL for protein expression in *E. coli*, primer pair #2946 and 2947 were used to amplify the *cotL* gene lacking its stop codon. The resulting PCR product was digested with Nde and XhoI and assembled into pET22b digested with the same enzymes using Gibson assembly. The assembly was transformed into DH5α, and the resulting construct was sequenced verified before transforming the plasmid into BL21(DE3) for protein expression.

**pET22b-CPD(TAA)**. To clone a His-tagged *Vibrio cholerae* cysteine protease domain (CPD) from the MARTX toxin gene, primer pair #2573 and 2574 were used to amplify the region encoding the CPD (aa 3439-3650) and to add a stop codon. The resulting PCR product was digested with NdeI and XhoI and Gibson assembled into pET22b digested with the same enzymes. The assembly was transformed into DH5α, and the resulting construct was sequenced verified before transforming the plasmid into BL21(DE3) for protein expression.

**pMTL-YN3 ∆*spoVQ***. Primer pair #2484 and 2486 were used to amplify the region 704 bp upstream of the *spoVQ* gene along with the first 22 codons off *C. difficile* genomic DNA. Primer pair #2485 and 2487 were used to amplify the region 665 bp downstream of the *spoVQ* gene and the last 12 codons of *spoVQ* using *C. difficile* genomic DNA as the template. The PCR products resulting PCR products were cloned into pMTL-YN3 digested with AscI and SbfI using Gibson assembly.

**pMTL-YN1C *spoVQ****.* To clone the *spoVQ* complementation construct, primer pair #2540 and 2543 was used to amplify the *spoVQ* gene including the stop codon and the region 232 bp upstream of *spoVQ*. The resulting PCR product was cloned into pMTL-YN1C digested with NotI and XhoI using Gibson assembly.

**pMTL-YN1C *spoVQ-FLAG_3_***. To clone the *spoVQ* complementation construct encoding a C-terminal FLAG_3_ epitope tag, primer pair #2540 and 2544 was used to amplify the *spoVQ* gene without the stop codon and its promoter region (232 bp upstream) along with sequence encoding part of a FLAG epitope. The resulting PCR product was assembled with the following g-block encoding the FLAG_3_ epitope into pMTL-YN1C along with digested with NotI and XhoI using Gibson assembly.

gBlock: Cdif CD3457-3xFLAG for pMTL-YN1C

GTATAGAAATGGACAAAATAGAAAGTGTGGTTGAGGAAGGAAAGGATGTTTTGAGAGTCAAGATAAAGTATAAAGATAAGGAAGATTCCTTTCCATACATAGTCGTTGAAACAAATATGAGTGAACTTCCAGATAGAATAGAATTAAATGCAACGAAAGATTATAAAGATGATGATGATAAAGACTATAAAGATGACGATGATAAGGATTATAAGGATGATGATGACAAATAACTCGAGGCCTGCAGACATGCAAGCTTGGCACTG

**pMTL-YN1C *spoVQ-mCherry***. To generate a construct encoding a C-terminal mCherry fusion to SpoVQ, primer pair #2540 and 2541 was used to amplify *spoVQ* and its promoter region (232 bp upstream) along with part of the *mCherry* gene*.* Primer pair #2542 and #2133 was used to amplify a codon-optimized *mCherry* gene as the template (8). The resulting PCR products were gel purified and assembled into pMTL-YN1C using Gibson assembly.

**pMTL-YN1C *spoVQ*_∆32_**. To generate a construct encoding a SpoVQ lacking its transmembrane domain spanning aa 1-32, primer pair #2540 and 2549 was used to amplify *spoVQ* and its promoter region (232 bp upstream) along with part of the *mCherry* gene*.* Primer pair #2550 and #2543 was used to amplify *spoVQ* (spanning codons 33 through its stop codon). The resulting PCR products were assembled into pMTL-YN1C using Gibson assembly.

**pMTL-YN3 ∆*cotL***. Primer pair #2820 and 2822 were used to amplify the region 763 bp upstream of the *cotL* gene along with the first 12 codons off *C. difficile* genomic DNA. Primer pair #2821 and 2823 were used to amplify the region 813 bp downstream of the *cotL* gene and the last 9 codons of *cotL* using *C. difficile* genomic DNA as the template. The PCR products resulting PCR products were cloned into pMTL-YN3 digested with AscI and SbfI using Gibson assembly.

**pMTL-YN1C *cotL****.* To clone the *cotL* complementation construct, primer pair #2833 and 2834 was used to amplify the *cotL* gene including the stop codon and the region 327 bp upstream of *cotL*. The resulting PCR product was cloned into pMTL-YN1C digested with NotI and XhoI using Gibson assembly.

**pMTL-YN1C *mCherry-sipL***. To generate a construct encoding an N-terminal mCherry fusion to SipL, primer pair #2165 and 3022 was used to amplify the promoter region of *sipL.* Primer pair #3021 and #3020 was used to amplify a codon-optimized *mCherry* gene as the template (8). Primer pair #3019 and 2166 was used to amplify the *sipL* gene including the stop codon off *C. difficile* genomic DNA. The resulting PCR products were gel purified and assembled into pMTL-YN1C using Gibson assembly.
